# Control of enzyme reactions by a reconfigurable DNA nanovault

**DOI:** 10.1038/s41467-017-01072-8

**Published:** 2017-10-19

**Authors:** Guido Grossi, Mette Dalgaard Ebbesen Jepsen, Jørgen Kjems, Ebbe Sloth Andersen

**Affiliations:** Interdisciplinary Nanoscience Center, Department of Molecular Biology and Genetics, Aarhus University, 8000 Aarhus C, Denmark

## Abstract

Biological systems use compartmentalisation as a general strategy to control enzymatic reactions by precisely regulating enzyme–substrate interactions. With the advent of DNA nanotechnology, it has become possible to rationally design DNA-based nano-containers with programmable structural and dynamic properties. These DNA nanostructures have been used to cage enzymes, but control over enzyme–substrate interactions using a dynamic DNA nanostructure has not been achieved yet. Here we introduce a DNA origami device that functions as a nanoscale vault: an enzyme is loaded in an isolated cavity and the access to free substrate molecules is controlled by a multi-lock mechanism. The DNA vault is characterised for features such as reversible opening/closing, cargo loading and wall porosity, and is shown to control the enzymatic reaction catalysed by an encapsulated protease. The DNA vault represents a general concept to control enzyme–substrate interactions by inducing conformational changes in a rationally designed DNA nanodevice.

## Abstract (web summary)

DNA nanostructures can cage enzymes but currently fall short of controlling their reactions with substrates. Here, the authors enclose an enzyme inside a dynamic DNA vault, which regulates its access to substrate molecules—and thus its enzymatic activity—through a multi-lock mechanism.

## Introduction

The ability to control single enzymes—defined as the ability to inactivate and activate a specific enzyme in a user-controlled fashion—has great interest in basic research, as well as in application-focused fields, such as biotechnology, synthetic biology, nanomedicine and bio-organic synthesis. DNA nanotechnology allows rational design of nanostructures and devices^1^, and represents a promising approach to organise and control enzymes for several reasons. First, DNA strands can be self-assembled into well-defined DNA nanostructures that can be used to spatially organise several different molecular components^2^. Second, enzymes are easily conjugated to DNA strands through several different methods and can be brought specifically together by base pair complementarity^3^. Third, DNA strand displacement can be used to dynamically assemble and disassemble DNA strands or reconfigure a DNA device between several states^4^. DNA nanostructures have been extensively employed to improve the yield of enzymatic cascades by scaffolding multiple enzymes^5–8^. On the other hand, only few systems to control single enzymes have been presented so far, mostly relying on the controlled interaction between the enzyme of interest and a coenzyme^9, 10^, an inhibitor^11, 12^ or a separated subdomain of the enzyme itself^13, 14^. However, these strategies have limited use since they depend on specific properties of the enzymes, and a general method for controlling single enzymatic activities is still missing.

In living organisms compartmentalisation is widely used to gate physical interactions between enzymes and their substrates^15^ and results in high local concentrations of specific enzymes that enhance reaction kinetics, channel metabolic substrates through enzymatic cascades, and sequester intermediates, thus reducing potential cell toxicity and competing cross-reactions^16, 17^. Membrane-bound organelles are the most common ways to compartmentalise enzymatic reactions in living organisms, but other varieties of nanoscale compartments delimited by proteins exist such as encapsulin shells^18^, carboxysomes^19^, vault particles^20^ and virus capsids. Such protein structures are capable of fuelling the activity of the enclosed enzymes by both passively importing specific substrates through selectively permeable shell proteins^21^, and by dynamically controlling the opening of proteic pores that attract and internalise small substrates molecules within the compartment^22^.

Taking inspiration from nature, scientists have developed artificial enzyme compartmental systems including lipid vessels^23^, polymersomes^24^, polymer capsules^25^ and protein cages^26, 27^. Similarly, rationally designed DNA nano-containers have been constructed characterised by solid walls, with the potential to fully enclose large biomolecules^28–30^. The first designed DNA nano-containers were originally envisioned for controlling the catalytic activity of encapsulated enzymes by regulating their access to relevant substrates^28^, but the experimental implementation of such a system has proven to be difficult. For example, DNA nanostructures have been used to cage enzymes, but without achieving control over their catalytic activity^31–34^. In other cases, DNA origami devices have used physical shielding to control the access to enclosed antibody fragments^35^ and DNAzymes^36^, but the lack of large and isolated cavities prevent strict control over enzymatic activities.

**Fig. 1:**
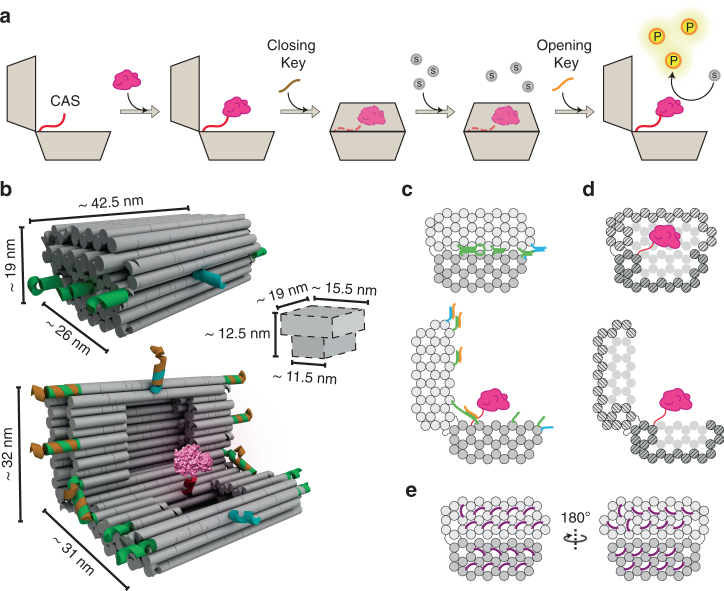
Working mechanism and structural design of the DNA Vault. **a** The open nanostructure is loaded with the enzyme (pink) at the cargo-anchoring site (CAS, red) and then closed by adding a sequence-specific closing key (brown). When the substrate (grey circles) is added, the encapsulated enzyme is inhibited from substrate interaction, unless a sequence-specific opening key (orange) is present in solution. Product (yellow circles) is now generated. **b** Three-dimensional rendering and size of the DNA Vault (DV) in the closed and open states. Cavity of closed structure is schematized. The front lock (cyan), side locks (green), CAS (red) and opening key (orange) are shown. The enzyme shown in pink is bovine alpha-chymotrypsin (aCt, PDB id: 1YPH). **c** Schematic side-view of enzyme-loaded DV in the closed (top) and open (bottom) states. The DNA helices are visualised as circles. Elements have same colour as in **b**. **d** Schematic section-view showing the inner cavity of the enzyme-loaded DV in the closed (top) and open (bottom) states. **e** End-views showing positions of staple loops (not shown in **b** for clarity) that cover holes in honeycomb lattice

Inspired by the general method of compartmentalisation, we conceive the idea of a DNA nanostructure that can control reactions catalysed by a single enzyme, by enclosing it within an internal cavity and exposing it to free substrate molecules only in response to specific molecular cues (Fig. 1a). We name this nanostructure the ‘DNA Vault’ (DV) because it is constructed to have solid walls and a multi-lock system to protect the encapsulated enzyme. Here we present the design of a 3D DNA origami nanodevice, characterize its reversible opening/closing mechanism, and load it with cargos of different chemo-physical properties. Furthermore, we develop an assay to characterize the permeability of DNA nano-containers, and use the DV to control the activity of an encapsulated enzyme. In conclusion, we successfully developed a general approach to control enzyme–substrate interactions by inducing conformational changes in a dynamic DNA nanostructure.

## Results

### Design of the DNA Vault

The DV was designed using the scaffolded DNA origami method^37^ based on the honeycomb lattice^38^ (Supplementary Tables 1 and 2). The structure is composed of two halves with dimensions of 26 × 42.5 × 9.5 nm^3^ and 22 × 42.5 × 9.5 nm^3^, respectively, that are connected through a hinge, and have an inner cavity of 3.2 zeptoliter (Fig. 1b; Supplementary Fig. 1). Close-packing of helices^39^ was used to create a tight interface between the two halves of the DV in the closed state (Fig. 1c, top). To control the opening and closing of the DV, five double-helix locks^28^ were placed on the two sides and front, and two hairpin locks^40^ were placed close to the hinge (Fig. 1b, c; Supplementary Fig. 2; Supplementary Note 1). The locks function by toehold-mediated DNA strand displacement^41, 42^, and are designed to be reversibly opened and closed by the addition of an opening key (OpK) and a closing key (ClK), respectively (Supplementary Figs. 3 and 4). A single-stranded DNA inside the cavity functions as a cargo-anchoring site (CAS) for enzyme loading (Fig. 1d). Finally, the ends of the helices on the outside of the DV are decorated with 28-nucleotide (nt) poly-T loops (Fig. 1e) to reduce aggregation caused by inter-molecular stacking interactions and to additionally decrease permeability of the side-walls^43^.

The nanostructures were assembled and analysed by gel electrophoresis (Supplementary Fig. 5) showing that ~50% of the self-assembled structures are in the monomeric form while the remaining part forms dimers or higher-order aggregates, which is commonly observed for compact 3D DNA origami structures^38^. Moreover, transmission electron microscopy (TEM) images of the closed DV (Supplementary Fig. 6b) indicate that ~65% of the monomeric structures are correctly folded while 35% show visible deformities that may not allow a correct function of the DV. Thus, we estimate that approximately 30% of all the self-assembled DNA nanostructures are correctly folded monomers. This estimate does not take into consideration minor structural errors such as missing staple strands, that are not distinguishable in TEM images, and could also affect the function of the DV.

The dimensions of closed and open structures were measured by TEM. In the closed sample, monomers were identified with back-to-front length of 26.0 ± 2.4 nm (Supplementary Fig. 7c), matching the theoretical value of 26 nm (Fig. 1b). In the open sample a back-to-front length of 30.5 ± 2.7 nm was measured (Supplementary Fig. 7d), matching the theoretical value of 31 nm which is expected for a structure with a tight hinge designed to open to an angle of 90° (Fig. 1b).

### Opening and closing mechanism

**Fig. 2:**
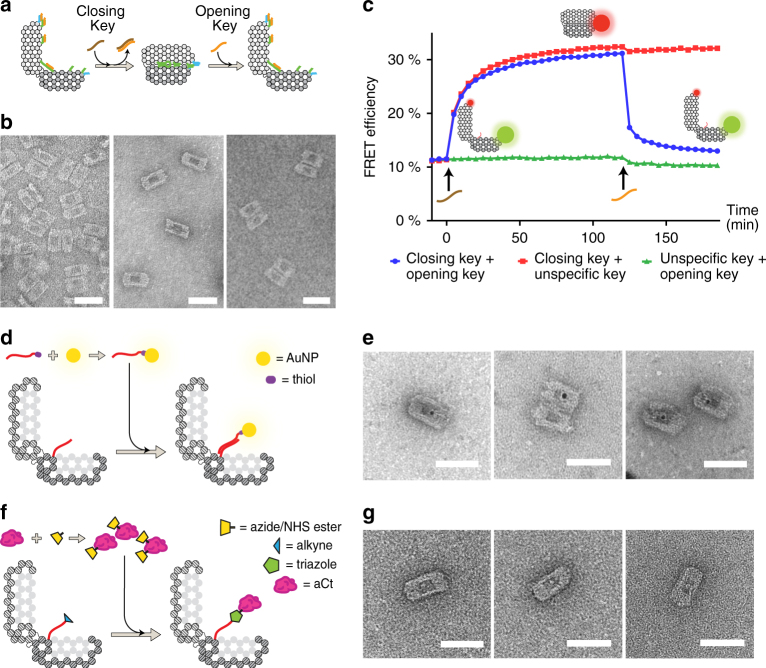
Reversible closing–opening mechanism and cargo loading. **a** Schematic of the closing–opening mechanism of the DNA nanostructure. **b** Transmission electron microscopy (TEM) images showing open, closed and reopened DV samples. Original TEM images in Supplementary Fig. 6. **c** FRET efficiency measured on the open Cy3/Cy5-conjugated DV. The blue line shows values measured after the closing key is added (brown), and—subsequently—after the opening key is added (orange). **d** Schematics of non-covalent cargo-loading mechanism: 5 nm gold nanoparticles (AuNPs) were reacted with thiol-modified DNA strands and incubated together with open DV molecules exposing a complementary CAS staple strand within the cavity. **e** TEM images of AuNP-loaded DV. AuNPs are detectable as black dots within the DV cavity. Original TEM images in Supplementary Fig. 10a–c. **f** Schematics of covalent cargo-loading mechanism: the endopeptidase aCt was conjugated with azide-NHS ester handles, and then reacted together with open DV exposing an alkyne-modified CAS staple strand within the cavity. Copper(I)-catalysed alkyne-azide cycloaddition reaction induces the formation of covalent DNA origami-enzyme conjugates. **g** TEM images of the aCt-loaded DV after incubation with closing key. Enzymes are detectable as white spots within the DV cavity. Original TEM images in Supplementary Fig. 10d–f. Scale bar, 50 nm

In order to encapsulate a native enzyme and control its activity the DV structure must have a flexible hinge to be able to reversibly close and open (Fig. 2a)^44^. The functionality of the hinge was investigated by labelling the two halves of the DV with Cy3 and Cy5 fluorophores and measuring Förster resonance energy transfer (FRET) upon addition of both OpK and ClK. It was found that an 8-nt hinge is required to close the structure efficiently (Supplementary Note 2; Supplementary Figs. 7b and 8). The DV with 8-nt hinge was assembled in the open state and investigated by TEM (Fig. 2b): the structures are observed in an open state showing the two halves of the DV adhering to the TEM grid (Fig. 2b, left). After addition of ClK the TEM imaging reveals closed structures with clear staining and contrast of the inner cavity (Fig. 2b, middle). Finally, after addition of OpK the TEM imaging reveals fully opened structures similar to the original sample (Fig. 2b, right). A FRET time-course experiment was performed starting from open DV with FRET efficiency of 11% (Fig. 2c). Upon addition of a 1.3-fold excess of ClK the value increased to 30% indicating device closure (blue line). After stabilisation of the FRET signal a 5-fold excess of OpK was added, which resulted in a fast decrease in FRET efficiency to 13%. The reason why the initial and final FRET values are not approaching 0% could be explained by the presence of DV aggregates in the sample. The opening and closing mechanism is demonstrated to respond only to specific DNA keys (Fig. 2c, green and red lines) and can be repeated multiple times (Supplementary Fig. 9).

### Cargo loading

To test packaging of cargos two different strategies were employed: non-covalent, annealing-driven loading of DNA-modified gold nanoparticles (AuNPs), and covalent bond formation between alkyne-exposing CAS and azide-modified enzyme via copper(I)-catalysed alkyne-azide cycloaddition (CuAAC). Initially, AuNPs of 5 nm in diameter were conjugated with 1:1 molar ratio of thiol-modified DNA strands with complementary sequence to the CAS staple strand, and then incubated together with open DV (Fig. 2d). TEM images of the sample revealed a single AuNP located within the cavity of 10–15% of the DNA nanostructures (Fig. 2e, Supplementary Fig. 10a–c). Next, the endopeptidase alpha-Chymotrypsin (aCt) of ~4.4 × 4.7 × 5.1 nm^3^ in size (PDB id 1YPH) was conjugated with azide handles. In order to exploit the most generally applicable strategy to modify proteins, we reacted commercially available wild-type aCt with NHS ester-azide heterobifunctional cross-linkers. These cross-linkers attach to exposed lysine residues on the surface of the target protein, and can therefore be used on virtually any soluble protein^6, 31, 45^. The resulting azide-modified aCt (Supplementary Fig. 11), was then reacted with open DV assembled with alkyne-modified single-stranded CAS module (DV-ssAlk; Fig. 2f). Enzyme-loaded DNA origami nanostructures were then closed and analysed by TEM, showing a single aCt housed within the cavity of 5–10% of the nanostructures (Fig. 2g, Supplementary Fig. 10d–f). The catalytic activity was partially retained upon chemical modification and packaging (Supplementary Note 3).

### Permeability of the DNA Vault

**Fig. 3:**
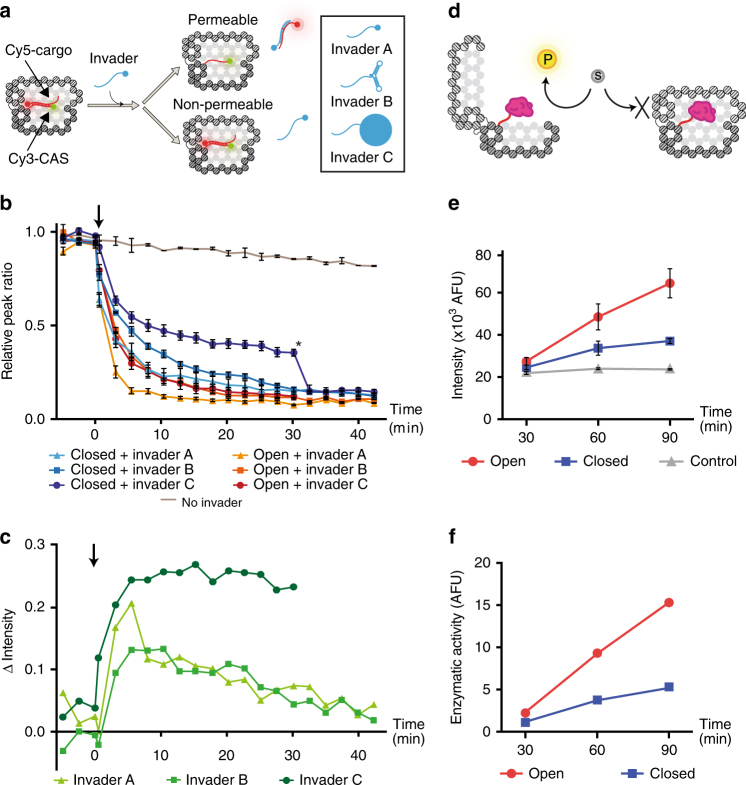
Permeability assay of the DNA Vault and enzyme activity control. **a** The DV encloses a fluorescent cargo consisting of a Cy5-modifed DNA strand annealed onto the Cy3-modified CAS strand, and exposing a single-stranded toehold region. When an invader is added, it can either penetrate into the nanostructure displacing the cargo, or it is shielded from the cavity, in which case the cargo is unaffected and remains within the DV. Three invader strands of different size are used: simple DNA strand (Invader A), dumbbell DNA strand (Invader B), and Streptavidin-bound DNA strand (Invader C). **b** Fluorescence data showing the decrease of relative peak ratio upon invader strand addition. Invader strands were added at 0 min and the fluorescence immediately measured afterwards. The black arrow indicates addition of invaders; the asterisk indicates addition of Invader A to Closed + Invader C after 30 min to confirm active shielding of the enclosed cargo from free-diffusing Invader C molecules. Bars indicate one standard error from the mean fluorescence signal as recorded in either three (Invader B) or four (Invader A and C) replicates. **c** Quantification of the shielding effect of the DV calculated as the difference of intensity values between closed and open samples, as shown in panel **b**. The black arrow indicates addition of invaders. **d** The proteolytic enzyme aCt (pink) is encapsulated inside the DV, while the substrate (grey circle) is free in solution. The enzyme catalyses the conversion of substrate to product (yellow circle) in the open structure, while the reaction is inhibited in the closed DV. **e** aCt enzymatic activity measured on open aCt-loaded (blue), closed aCt-loaded (red) and open empty DV (control, grey). Bars indicate one standard error from the mean fluorescence signal as recorded in three replicates. **f**, Normalisation of measured enzymatic activity as in **e** after control subtraction (closed—control at 30 min = 1)

To assess the ability of the DV to shield the internal cavity from free-diffusing molecules, a permeability assay was performed (Fig. 3a). The DV was assembled in the open state with a fluorescent CAS module consisting of the Cy3-modified CAS strand annealed to a Cy5-modified DNA cargo strand exposing a single-stranded toehold. The proximity of the Cy3 and Cy5 fluorophores on the CAS is within FRET distance. In order to measure the molecular permeability of the DNA walls of the nanostructure, open and closed DV were incubated with three invaders of different size: Invader A, a short single-stranded DNA; Invader B, a DNA strand with dumbbell secondary structure, ~4 nm long, and Invader C, a DNA strand bound to streptavidin of ~5 nm in diameter^46^. The invaders were designed to displace the Cy5-modified cargo from the Cy3-modified CAS leading to separation of the dyes and loss of FRET. Displacement experiments performed on the CAS/cargo system alone (Supplementary Fig. 12) demonstrate that the difference in size and chemo-physical properties of the invader strands does not affect the cargo displacement process significantly. The cargo displacement process in the DV was observed by measuring changes in the intensity of the Cy3 and Cy5 peaks and calculating the ratio between them (Fig. 3b). Fast displacement of the cargo is observed when any of the invaders is added to the open structure (from 0.95 ± 0.02 to 0.10 ± 0.01) with Invader A having the faster kinetics. Contrarily, slower kinetics are observed when the closed nanostructure is challenged with the invaders (where slower displacement rates are correlated with bigger invaders), showing that the closed DV is shielding the cargo from the invaders. Initially, a fast decrease in cargo signal is observed indicating that a fraction of the DV sample does not shield the cargo efficiently, which can be explained by the presence of malformed structures in the sample, as indicated by the previously mentioned yield analysis. Nevertheless, well-folded structures with a persistent shielding effect are observed when Invader C is added to the closed DV, where the cargo signal stabilizes at a value of 0.35 ± 0.02. This effect is confirmed by adding Invader A to the reaction (star in Fig. 3b), resulting in further decrease of the cargo signal to baseline levels, which indicates that the closed DV shields the cargo from Invader C, but not from the smaller Invader A.

In order to quantify the shielding efficiency further, we calculated the difference between the cargo displaced from open and closed nanostructures (Δ*I*). The resulting curves are characterised by two kinetic steps (Fig. 3c). When either Invader A or Invader B are used, Δ*I* quickly increases at first, indicating that the closed DV is protecting the internal cargo from the invaders. However, after 5 min of incubation, Δ*I* slowly decreases, suggesting that the invaders eventually penetrate inside the closed DV and displace the cargo from the cavity. However, when Invader C is added, Δ*I* quickly increases at first, and then stabilizes at around 0.25, suggesting that the closed DV persistently shields its internal cavity from particles of at least 5 nm in diameter.

### Control of enzyme–substrate interaction

Finally, we tested the effect of opening and closing the aCt-loaded DV on aCt activity by using the fluorogenic substrate fluorescein isothiocyanate (FITC)-casein with an estimated size of 4.0–4.1 nm in diameter (the size was calculated based on a molecular weight of 28–30 kDa as estimated by gel electrophoresis^47^) (Fig. 3d). Open DV-ssAlk nanostructures were loaded with azide-exposing aCt, purified from excess unconjugated enzyme (Supplementary Note 4 and Supplementary Fig. 13), and closed. The sample was then split in two tubes: one tube was incubated with correct OpK, the other with unspecific key. Finally, open and closed nanostructures were incubated with FITC-casein and analysed at specific time points by measuring fluorescence^48^. It was observed that the enzymatic activity measured on open aCt-loaded DV was up to 3 times higher than on closed nanostructures and increasing linearly during the time analysed (Fig. 3e, f). Proteolytic activity is also observed for the closed DV sample, which can be explained by the presence of incorrectly folded DV nanostructures identified by the yield analysis.

A double-stranded CAS was also tested in order to investigate the possibility to load DNA-conjugated enzymes into the DV by annealing. However, the measured difference between open and closed DV was less prominent compared to DV-ssAlk, suggesting that a bigger enzyme-anchor can affect the ability of the DV to close and thus decreases its shielding property (Supplementary Note 5 and Supplementary Fig. 14).

## Discussion

The current study provides proof-of-concept for enzyme control by shielding from or exposure to substrate using a dynamic DNA origami device. To achieve this goal, we have combined multiple design principles to construct an unstrained dynamic device with reversible opening/closing mechanism and a cavity with limited accessibility. The ability of the DV to physically hinder enzyme–substrate interactions was demonstrated to inhibit the enzymatic activity of an encapsulated protease. The approach should be generally applicable to several enzyme–substrate systems and we provide a protocol that with slight modification should allow the loading of any enzyme in the DV (Supplementary Note 6).

The DV was purified by polyethylene glycol (PEG) precipitation, which removes excess staple strands without separating well-folded monomers from aggregates. The yield of correctly folded monomeric DNA nanostructures was estimated to be ~30% based on gel electrophoresis and TEM analyses. This is also reflected by the permeability assay against the large invader, that shows a shielding effect of ~25%, which clearly indicates that this fraction efficiently shields the enclosed cargo over the timescale assayed. The yield of correctly folded structures can possibly be increased through further purification of monomers, or improved sequence design^49^ and self-assembly protocol for the DV.

The enzyme loading yield into well-folded structures was estimated by TEM to be 5–10%. The low yield could be due to unfavourable CuAAC reaction conditions in the constrained cavity of the DV. Another loading strategy that could improve the loading yield could be to conjugate a DNA strand to the enzyme and afterwards anneal it to the CAS of the open DV. However, we showed that a double-stranded CAS partially decreased the shielding function of the DV. Another drawback of the DNA-protein conjugation method used was that it partially inhibited the activity of aCt. The inhibition of enzyme activity may be avoided by using protein engineering^50, 51^ or DNA-templated chemistry^52^ to produce a site-specific DNA-protein conjugate. However, such approaches are often more time-consuming and less generally applicable.

The enzyme activity assay was performed by using the DV to restrict aCt access to its substrate FITC-casein, and showed that the enzymatic activity of the open sample was 3 times higher compared to the closed sample. This effect was higher than expected since the permeability assay showed that only 25% of the structures efficiently shield the cargo from Invader C, which has similar size as FITC-casein. If every DV contained an aCt cargo, only 1.25 times higher activity would be expected. Thus, we speculate that the loading procedure is biased towards enzyme packaging in well-folded open structures that expose the alkyne-modified CAS, and not into misfolded or aggregated structures that hide the CAS. This procedure differs from the cargo loading protocol used for the permeability assay, where the fluorescent cargo is heat-annealed during the self-assembly process, and is expected to be present on every DV structure. Additionally, the activity of the enzyme could be affected by the microenvironment of the DV particle. In fact, the close proximity of DNA to aCt inside the closed DV will decrease the pH in the proximity of the enzyme^53^ and thus lower the catalytic activity of aCt, which has a slightly basic optimum pH^54^.

In conclusion, the DNA Vault integrates the catalytic activity of natural enzymes with rationally designed DNA nanostructures, so that enzymes can be exposed to substrate in response to specific combinations of DNA signals^28, 35, 55^. Moreover, the modular locking system employed here can be modified in order to respond to the presence of proteins^35^, RNA sequences^56^ and small molecules^57^, as well as environmental variations in pH^58^, temperature^32^ and ion concentration^59^. Modified DNA vaults with higher assembly yields and lower permeability may be needed to control enzymes that act on smaller substrates. The porosity might be modified by addition of more DNA layers or structures such as entropic brushes, chemical compounds, protein shells and lipid membranes to the exterior of the device. Nevertheless, the general approach described here should allow a wide range of enzyme–substrate systems to be controlled. With further development, natural enzymes can be programmed to operate as signal amplifiers for diagnostics applications and as active components of delivery vehicles for advanced applications in medicine.

## Methods

### Materials

Chemicals were purchased from Sigma-Aldrich, unless otherwise noted. Standard desalted DNA oligonucleotides were purchased from Integrated DNA Technologies (IDT); oligonucleotides longer than 60 nt and those bearing chemical modifications were ordered as high-performance liquid chromatography-purified, all remaining staple strands were used without further purification.

### Design process

CaDNAno software^60^ was used as primary design tool, with honeycomb lattice. Sequences of the lock module were designed in Nupack^61^. AutoDesk Maya (http://www.autodesk.com) with CaDNAno plugin was used for 3D modelling and rendering of the structure.

### Self-assembly process

DNA origami self-assembly was performed by mixing scaffold ssDNA (M13mp18, Bayou Biolabs) with 5× molar excess of staple strands in TAE/Mg^2+^ buffer (40 mM Tris/Acetate, 1 mM EDTA, pH 8.3, 16 mM MgCl_2_). The assembly reaction was performed by incubating the samples at 75 °C for 15 min, followed by a temperature ramp of −0.1 °C/1.5 min to 60 °C, and afterward a ramp of −0.1 °C every 6 min to 20 °C.

### Structure purification

When required for experiments the DNA origami structure was purified from excess of staple strands by PEG precipitation, according to the literature^62^, unless noted otherwise.

### Closing and opening

Open DV is closed by adding MgCl_2_ to 30 mM final concentration and ClK at lock: ClK = 1:1.3 times molar excess, and then incubating the sample at room temperature for 2 h and subsequently at 4 °C for 36 h, unless noted otherwise. Closed DV is opened by adding OpK at ClK: OpK = 1:5 times molar excess and incubating the sample at room temperature for 1 h, unless noted otherwise.

### Agarose gel analysis

Annealed DNA structures were directly loaded on 1.5% agarose gel (if not otherwise indicated) and allowed to migrate for 3 h at 4 °C (running buffer: 1X TBE, 10 mM MgCl_2_; running voltage: 80 V). The gel was stained with SybrGold (Life Technologies) and visualised with Typhoon Trio scanner (GE Healthcare Life Sciences).

### TEM imaging

Unpurified samples were deposited on glow-discharged 200 mesh carbon-coated copper TEM grids (Ted Pella Inc.) for 1.5 min, and positively stained with 1% uranyl acetate solution for 1 minute. A TEM FEI Tecnai G2 Spirit (Bio)twin was used at either 80-kV or 120 kV acceleration voltage and images were recorded and analysed with either RADIUS software (Olympus) or EM-Menu4 (TVIPS). Statistical analyses of the DNA origami size (Supplementary Fig. 7c, d) were executed with PAST 2.07 software (http://folk.uio.no/ohammer/past)^63^.

### FRET analysis

The DV was assembled with a 3′-Cy3-modified and a 5′-Cy5-modified staple strand purchased from IDT. Closed and open DV samples were purified from unincorporated staple strands and analysed with FluoroMax-3 fluorometer (Horiba Scientific). Samples were excited at 530 nm (excitation of Cy3) and 600 nm (excitation of Cy5), with entrance slit set to 5 nm, and integration time set to 0.5 s. Emission was recorded at 565 nm and 666 nm for Cy3 and Cy5, respectively. All the experiments were conducted at 25 °C constant temperature. FRET efficiency values were calculated as *E* = *I*_DA_/(*I*_DA_ + *I*_DD_), where *I*_DD_ and *I*_DA_ are the Cy3 and Cy5 emission intensities, respectively, upon donor excitation.

### Cargo displacement experiments

Biotinylated Invader A was incubated with streptavidin in 1:2 molar ratio for 15 min at room temperature, then used as Invader C without further purification. Native PAGE analysis shows no unreacted biotinylated Invader A (data not shown). Closed purified DV was incubated with either OpK or unspecific key at RT for 30 min. Open or closed cargo-loaded DV were then analysed with FluoroMax-4 fluorometer (Horiba Scientific), and incubated with invader strands in 10 times molar excess at room temperature. Samples were excited at 530 nm (excitation of Cy3) and 600 nm (excitation of Cy5), with entrance slit set to 5 nm, and integration time set to 0.5 s. Emission was recorded at 565 nm and 666 nm for Cy3 and Cy5, respectively. All the experiments were conducted at room temperature. Relative peak ratios were calculated using the formula *I*_DA_/(*I*_DA_ + *I*_DD_), where *I*_DD_ and *I*_DA_ are the Cy3 and Cy5 emission intensities, respectively, upon donor excitation. The minimum relative peak ratio was determined on a sample containing a Cy3-modified and a Cy5-modified oligonucleotides (50[121]-(3′)Cy3 and 27[117]-(5′)Cy5 from Supplementary Table 2, respectively) that do not interact with each other. The maximum relative peak ratio was determined as the highest value measured on DV before adding any invader molecule.

### AuNP-oligonucleotide conjugation and AuNP loading

5-nm AuNPs were purchased from Ted Pella Inc., thiol-modified oligonucleotides from IDT. Bis(p-sulfonatophenyl)-phenylphosphine-dihydrate-dipotassium salt (phosphine, 1 mg) was added to 5 ml AuNPs, the mix was covered with aluminium foil and stirred slowly for 24 h. NaCl was added until the colour changed from red to bluish, then the mix was centrifuged at 750 g for 15 min. The pellet was resuspended in 100 µl of 2.5 mM phosphine and 250 µl methanol was added. The mix was centrifuged at 750 g for 15 min, and the pellet resuspended in 100 µl of 2.5 mM phosphine (this is the phosphinated AuNP solution). Concentration of the phosphinated AuNPs was calculated with Nanodrop 1000 spectrophotometer (Thermo Scientific), then 1:1 molar ratio of thiol-modified oligonucleotide and phosphinated AuNPs were combined with TBE (0.25× final concentration) and NaCl (50 mM final concentration), and shaken on a horizontal shaker at 900 rpm for 24 h. DNA-conjugated AuNPs were loaded on 3% agarose gel (run for 2 h at 100 V) in order to detect a gel mobility shift compared to unconjugated phosphinated AuNPs. DNA-conjugated AuNPs were washed 3 times with 0.5× TBE by using Amicon Ultra-0.5 ml centrifugal filters (50 kDa) to remove unconjugated oligonucleotides. AuNP concentration was finally calculated using Nanodrop, and then the obtained AuNP-DNA conjugates were incubated with the open DV in 10:1 molar ratio without further purification for 12 h at room temperature.

### aCt-oligonucleotide conjugation and enzyme loading

aCt was purchased from Sigma-Aldrich; NHS-C3-azide (BCL-014) and click reaction chemicals were a gift from baseclick GmbH. Enzyme-DNA conjugation was conducted in 2 steps: (i) enzyme conjugation with NHS-azide linker, and (ii) enzyme-azide CuAAC ‘click’ reaction with alkyne-modified oligonucleotide. First, aCt (14 lysine residues/molecule) was resuspended in aCt storage buffer (1 mM Tris/HCl, 2 mM CaCl_2_, pH 8.0) at 100 µM final concentration; NHS-azide linkers were resuspended in DMSO at 10 mM final concentration. aCt (2 nmol) was incubated with NHS-C3-azide linker in lysine: linker = 1:10 molar ratios, and shaken (400 rpm) at 21 °C for 2 h in 50 µl aCt storage buffer final volume. The sample was then washed 2 times with aCt storage buffer by using Amicon Ultra-0.5 ml centrifugal filters (50 kDa) to remove unreacted NHS-C3-azide molecules. Next, 25 pmol aCt-azide conjugates were reacted with alkyne-modified DNA strand in lysine: DNA = 1:2 molar ratio—by using Oligo-Click-S-Basic reaction kit (baseclick GmbH)—and shaken (500 rpm) at 25 °C for 1 h in 12 µl final volume. Enzyme-DNA conjugates were analysed by non-reducing 12% sodium dodecyl sulfate polyacrylamide gel electrophoresis (SDS-PAGE) to determine the DNA-conjugation yield. Enzyme concentration was calculated with NanoPhotometer P-300 spectrophotometer (Implen GmbH). 5 pmol purified alkyne-exposing DV were reacted with purified aCt-azide conjugates in 1:10 molar ratio by using Oligo-Click-S-Basic reaction kit (baseclick GmbH)—and shaken (500 rpm) at 25 °C for 1 h. The sample was split in 4 Oligo-Click-S-Basic reaction kit tubes for a final volume of 28.5 µl per tube. Excess enzyme was purified by using precipitation with PEG.

### SDS-PAGE analysis

Enzyme conjugates were run on denaturing non-reducing 12% polyacrylamide gel—by using Mini-PROTEAN System (Bio-Rad)—for 1 h at 200 V. Proteins were stained with Coomassie solution (0.125%) by shaking for 30 min at room temperature. Running gel buffer (1.5 M Tris/HCl, pH 8.8), stacking gel buffer (1 M Tris/HCl, pH 6.8), electrophoretic buffer (25 mM Tris, 250 mM Gly, 10% SDS), loading dye (0.6 M Tris/HCl, 40% glycerol, 8% SDS, 0.4 mg/ml bromophenol blue) were used.

### aCt enzymatic assays with sAAPFpNA

Chromogenic substrate N-succinyl-Ala-Ala-Pro-Phe-P-nitroanilide was purchased from Sigma-Aldrich and resuspended in DMSO at 20 mM final concentration. Enzymatic activity assays were performed by incubating 400 nM unmodified aCt, aCt-N3 or aCt-DNA in DV activity buffer (Buffer 1: TAE + 30 mM MgCl_2_ + 3 mM CaCl_2_), and in aCt storage buffer (Buffer 2: 1 mM Tris + 2 mM CaCl_2_), then adding 400 µM substrate in 10 µl total volume, mixing and quickly measuring absorbance at 405 nm of a 3 µl aliquot, by using NanoPhotometer P-300 spectrophotometer (Implen GmbH). Activity values were calculated as: (OD_15 s_—OD_0 s_)×4, where OD_15 s_ is the absorbance *A*_405 nm_ value after 15 s, and OD_0 s_ is the first absorbance *A*_405 nm_ value recorded.

### aCt enzymatic assays with FITC-Casein

FITC-casein was purchased as Pierce Fluorescent Protease Assay Kit (Thermo Fisher Scientific) and a slightly modified protocol was used. Briefly, 20 µl FITC-Casein (10×) were added to 20 µl DV sample (50 nM). The sample was gently mixed and incubated at 25 °C in the dark for 90 min maximum: 10 µl aliquots were taken at specific time points and added to 37.5 µl trichloroacetic acid (0.6 N). Samples were gently mixed, incubated at 25 °C in the dark for 30 min, and centrifuged for 10 min at 10,000 g. 10 µl of supernatant were added to 100 µl Tris (500 mM, pH 8.5) and gently mixed. The fluorescence intensity of FITC was finally recorded with FluoroMax-4 fluorometer (Horiba Scientific) (excitation = 485 nm; emission = 520 nm).

### Data availability

The authors declare that the main data supporting the findings of this study are available within the article and its Supplementary Information file. Extra data are available from the corresponding author upon request.

## Electronic supplementary material

Supplementary information

## References

[1] Seeman NC (2010). Nanomaterials based on DNA. Annu. Rev. Biochem..

[2] Zhang F, Nangreave J, Liu Y, Yan H (2014). Structural DNA nanotechnology: state of the art and future perspective. J. Am. Chem. Soc..

[3] Sacca B, Niemeyer CM (2011). Functionalization of DNA nanostructures with proteins. Chem. Soc. Rev..

[4] Zhang DY, Seelig G (2011). Dynamic DNA nanotechnology using strand-displacement reactions. Nat. Chem..

[5] Wilner OI (2009). Enzyme cascades activated on topologically programmed DNA scaffolds. Nat. Nanotechnol..

[6] Fu J (2014). Multi-enzyme complexes on DNA scaffolds capable of substrate channelling with an artificial swinging arm. Nat. Nanotechnol..

[7] Linko V, Eerikainen M, Kostiainen MA (2015). A modular DNA origami-based enzyme cascade nanoreactor. Chem. Commun..

[8] Fu Y (2013). Single-step rapid assembly of DNA origami nanostructures for addressable nanoscale bioreactors. J. Am. Chem. Soc..

[9] Freeman R, Sharon E, Tel-Vered R, Willner I (2009). Supramolecular cocaine− aptamer complexes activate biocatalytic cascades. J. Am. Chem. Soc..

[10] Liu M (2013). A DNA tweezer-actuated enzyme nanoreactor. Nat. Commun..

[11] Saghatelian G (2003). DNA detection and signal amplification via an engineering allosteric enzyme. J. Am. Chem. Soc..

[12] Gianneschi NC, Ghadiri MR (2007). Design of molecular logic devices based on a programmable DNA-regulated semisynthetic enzyme. Angew. Chem. Int. Ed. Engl..

[13] Erkelenz M, Kuo CH, Niemeyer CM (2011). DNA-mediated assembly of cytochrome P450 BM3 subdomains. J. Am. Chem. Soc..

[14] Sancho Oltra N, Bos J, Roelfes G (2010). Control over enzymatic activity by DNA‐directed split enzyme reassembly. Chembiochem.

[15] Martin W (2010). Evolutionary origins of metabolic compartmentalization in eukaryotes. Philos. Trans. R. Soc. Lond. B Biol. Sci..

[16] Kuchler A, Yoshimoto M, Luginbuhl S, Mavelli F, Walde P (2016). Enzymatic reactions in confined environments. Nat. Nanotechnol..

[17] Chen AH, Silver PA (2012). Designing biological compartmentalization. Trends Cell Biol..

[18] McHugh CA (2014). A virus capsid‐like nanocompartment that stores iron and protects bacteria from oxidative stress. EMBO J..

[19] Yeates TO, Kerfeld CA, Heinhorst S, Cannon GC, Shively JM (2008). Protein-based organelles in bacteria: carboxysomes and related microcompartments. Nat. Rev. Microbiol..

[20] Berger W, Steiner E, Grusch M, Elbling L, Micksche M (2009). Vaults and the major vault protein: novel roles in signal pathway regulation and immunity. Cell. Mol. Life Sci..

[21] Chowdhury C (2015). Selective molecular transport through the protein shell of a bacterial microcompartment organelle. Proc. Natl Acad. Sci. USA.

[22] Jacques DA (2016). HIV-1 uses dynamic capsid pores to import nucleotides and fuel encapsidated DNA synthesis. Nature.

[23] Walde P, Ichikawa S (2001). Enzymes inside lipid vesicles: preparation, reactivity and applications. Biomol. Eng..

[24] Nardin C, Widmer J, Winterhalter M, Meier W (2001). Amphiphilic block copolymer nanocontainers as bioreactors. Eur. Phys. J. E.

[25] Sakr OS, Borchard G (2013). Encapsulation of enzymes in Layer-by-Layer (LbL) structures: latest advances and applications. Biomacromolecules.

[26] Glasgow JE, Asensio MA, Jakobson CM, Francis MB, Tullman-Ercek D (2015). Influence of electrostatics on small molecule flux through a protein nanoreactor. ACS Synth. Biol..

[27] Comellas-Aragones M (2007). A virus-based single-enzyme nanoreactor. Nat. Nanotechnol..

[28] Andersen ES (2009). Self-assembly of a nanoscale DNA box with a controllable lid. Nature.

[29] Ke Y (2009). Scaffolded DNA origami of a DNA tetrahedron molecular container. Nano. Lett..

[30] Kuzuya A, Komiyama M (2009). Design and construction of a box-shaped 3D-DNA origami. Chem. Commun..

[31] Erben CM, Goodman RP, Turberfield AJ (2006). Single-molecule protein encapsulation in a rigid DNA cage. Angew. Chem..

[32] Juul S (2013). Temperature-controlled encapsulation and release of an active enzyme in the cavity of a self-assembled DNA nanocage. ACS Nano.

[33] Zhao Z (2016). Nanocaged enzymes with enhanced catalytic activity and increased stability against protease digestion. Nat. Commun..

[34] Sprengel A (2017). Tailored protein encapsulation into a DNA host using geometrically organized supramolecular interactions. Nat. Commun..

[35] Douglas SM, Bachelet I, Church GM (2012). A logic-gated nanorobot for targeted transport of molecular payloads. Science.

[36] Torelli E (2014). A DNA origami nanorobot controlled by nucleic acid hybridization. Small.

[37] Rothemund PW, Folding DNA (2006). to create nanoscale shapes and patterns. Nature.

[38] Douglas SM (2009). Self-assembly of DNA into nanoscale three-dimensional shapes. Nature.

[39] Ke Y, Voigt NV, Gothelf KV, Shih WM (2012). Multilayer DNA origami packed on hexagonal and hybrid lattices. J. Am. Chem. Soc..

[40] Zadegan RM (2012). Construction of a 4 zeptoliters switchable 3D DNA box origami. ACS Nano.

[41] Srinivas N (2013). On the biophysics and kinetics of toehold-mediated DNA strand displacement. Nucleic Acids Res..

[42] Yurke B, Turberfield AJ, Mills AP, Simmel FC, Neumann JL (2000). A DNA-fuelled molecular machine made of DNA. Nature.

[43] Kegler K (2008). Polyelectrolyte-compression forces between spherical DNA brushes. Phys. Rev. Lett..

[44] Marras AE, Zhou L, Su HJ, Castro CE (2015). Programmable motion of DNA origami mechanisms. Proc. Natl Acad. Sci. USA.

[45] Niemeyer CM, Sano T, Smith CL, Cantor CR (1994). Oligonucleotide-directed self-assembly of proteins: semisynthetic DNA—streptavidin hybrid molecules as connectors for the generation of macroscopic arrays and the construction of supramolecular bioconjugates. Nucleic Acids Res..

[46] Hyre DE (2006). Cooperative hydrogen bond interactions in the streptavidin-biotin system. Protein Sci..

[47] Erickson HP (2009). Size and shape of protein molecules at the nanometer level determined by sedimentation, gel filtration, and electron microscopy. Biol. Proced. Online.

[48] Twining SS (1984). Fluorescein isothiocyanate-labeled casein assay for proteolytic enzymes. Anal. Biochem..

[49] Dunn KE (2015). Guiding the folding pathway of DNA origami. Nature.

[50] Sacca B (2010). Orthogonal protein decoration of DNA origami. Angew. Chem. Int. Ed. Engl..

[51] Johnson JA, Lu YY, Van Deventer JA, Tirrell DA (2010). Residue-specific incorporation of non-canonical amino acids into proteins: recent developments and applications. Curr. Opin. Chem. Biol..

[52] Rosen CB (2014). Template-directed covalent conjugation of DNA to native antibodies, transferrin and other metal-binding proteins. Nat. Chem..

[53] Zhang T, Tsitkov S, Hess H (2016). Proximity does not contribute to activity enhancement in the glucose oxidase–horseradish peroxidase cascade. Nat. Commun..

[54] Kaspar P, Moller G, Wahlefeld A (1984). New photometric assay for chymotrypsin in stool. Clin. Chem..

[55] Zadegan RM, Jepsen MD, Hildebrandt LL, Birkedal V, Kjems J (2015). Construction of a fuzzy and Boolean logic gates based on DNA. Small.

[56] Zhong H, Seeman NC (2006). RNA used to control a DNA rotary nanomachine. Nano. Lett..

[57] Kuzuya A, Sakai Y, Yamazaki T, Xu Y, Komiyama M (2011). Nanomechanical DNA origami ‘single-molecule beacons’ directly imaged by atomic force microscopy. Nat. Commun..

[58] Liu Z, Li Y, Tian C, Mao C (2013). A smart DNA tetrahedron that isothermally assembles or dissociates in response to the solution pH value changes. Biomacromolecules.

[59] Gerling T, Wagenbauer KF, Neuner A, Dietz H (2015). 71 Dynamic DNA devices and assemblies formed by shape-complementary, non-basepairing 3D components. J. Biomol. Struct. Dyn..

[60] Douglas SM (2009). Rapid prototyping of 3D DNA-origami shapes with caDNAno. Nucleic Acids Res..

[61] Zadeh JN (2011). NUPACK: analysis and design of nucleic acid systems. J. Comput. Chem..

[62] Stahl E, Martin TG, Praetorius F, Dietz H (2014). Facile and scalable preparation of pure and dense DNA origami solutions. Angew. Chem. Int. Ed. Engl..

[63] Hammer O, Harper DAT, Ruyan PD (2001). PAST: paleontological statistics software package for education and data analysis. Palaentologia Electron..

