# Supplementary material for: Control of enzyme reactions by a reconfigurable DNA nanovault
Source: Nat Commun. 2017 Oct 19;8:992. doi: 10.1038/s41467-017-01072-8 (PMC5648847; doi:10.1038/s41467-017-01072-8)
Supplement: Supplementary file 1 — Supplementary information [file 41467_2017_1072_MOESM1_ESM.pdf]

Supplementary Note 1. Design of hinges and locks.

The DV hinge module is composed of 5 staple strands that bridge the two halves of the DV connecting the DNA helices 37 and 39 (Supplementary Fig. 2a,b). These hinge strands protrude from the DNA helices pointing approximately at each other in order to define a tight hinge region in DV-H1 (see main text and Supplementary Note 2). The DV locking system is designed to mimic a zip closure, where the stem-loop locks initiates the closing mechanism to move the sets of double-helix locks closer to the hinge region (the rear locks) into closer proximity. The rear locks will then interact forcing the two halves of the DV even closer together, thus facilitating the interaction between the next sets of locks: the side locks farther from the hinge (the middle locks) and the front lock. Finally, the middle locks and the front lock will anneal stabilizing the DV in a tightly closed conformation.

The position and orientation of both stem-loop and double-helix lock staple strands were designed in order to force the two halves of the DV as close as possible. For this purpose we used two different and complementary design approaches. The stem-loop lock strands (Supplementary Fig. 2a,c) and the rear double-helix lock strands (Supplementary Fig. 2a,d) were designed to protrude from the DNA helices pointing at each other in order to favour lock-to-lock interactions during the first steps of the closing mechanism. The double-helix middle lock strands (Supplementary Fig. 2a,e) and the front lock strands (Supplementary Fig. 2a,f) instead were designed to protrude pointing in the same direction in order to force the DNA helices they protrude from closer together in the end. The obtained effect is that the DNA helices at the interface of the two halves of the DV should adopt the tightly packed hexagonal lattice.

Supplementary Note 2. Characterization of the hinge region.

When DV-H1 was assembled in the closed state it migrated as one major band in gel electrophoresis (Supplementary Fig. 7a), which was observed in TEM images to be composed mainly of dimers (Supplementary Fig. 7c). Opening the DV by incubation with OpK resulted in a faster mobility species in gel electrophoresis (Supplementary Fig. 7a) and the observation of monomeric structures in TEM images (Supplementary Fig. 7d).

To investigate the opening mechanism in real time we labelled the two halves of the DV with Cy3 and Cy5 fluorophores, respectively, to allow the monitoring of the closed/open conformation by Förster resonance energy transfer (FRET). FRET measurements revealed low efficiency values for a closed nanostructure (10%; Supplementary Fig. 7b). This is probably because DV that form dimers are not correctly closed: double-helix lock staples that interact with lock staples belonging to different structures are not available to interact with the lock staples belonging to the same structure, thus not keeping the DV closed. Nevertheless, when OpK is added FRET efficiency values decrease from 10% to 5% indicating that Cy3 and Cy5 molecules are moving apart. This could be due to a combination of opening of the DV structure, and to the disruption of the dimerization interactions that cause DV monomers to fall apart.

The FRET analysis did not allow a satisfying characterization of the opening/closing mechanism of DV-H1. Therefore two different strategies were adopted to obtain a better understanding of the nanomechanical properties of the DV: (i) we directly measured the size from TEM images of DV assembled in the closed state and from DV incubated with OpK, and (ii) we measured FRET efficiency on DV assembled in the open conformation after incubation with ClK. First, in order to understand if the DV with stiff hinge is capable of opening we measured the size of 100 monodispersed structures assembled in the closed state, and after incubation with OpK, as visualized on TEM (Supplementary Fig. 7c). The statistical analysis conducted shows a value of

26.0 ± 2.4 nm for the closed structure (theoretical value = 26 nm), and a value of 30.5 ± 2.7 nm for the open structure (theoretical value = 31 nm) indicating that DV monomers are successfully assembled in the closed state, and that they open upon incubation with 5-fold molar excess of OpK. Next, we assembled Cy3/Cy5-modified DV-H1 in the open conformation and analysed it for FRET efficiency. The FRET efficiency values measured are consistent with the previous analysis: 5% FRET efficiency was measured on the open DV, increasing to 10% after incubation with ClK (Supplementary Fig. 8, blue line), thus demonstrating that DV with stiff hinge closes upon incubation with 1.3 fold molar excess of ClK.

These structural observations can be explained by a tight hinge region of DV-H1, which would lead to decreased DV closure allowing the locks to dimerize and to the tight 90-degree open conformation observed. To investigate this hypothesis we designed two more DV structures with different length of the hinge region of 4 nt (DV-H2) and 8 nt (DV-H3) to gradually increase the flexibility of the hinge. The structures were assembled in the open state and had similar gel mobility (data not shown) and low FRET efficiency as DV-H1 (Supplementary Fig. 8). However, the three DV behave differently when the ClK was added: the FRET efficiency value rose only 5% for DV-H1, whereas it rose 12% for DV-H2 and 20% for DV-H3. This clearly shows that a flexible hinge of 8 nt is required to allow closure of the DV structure, while a tight hinge region inhibits correct closure.

Supplementary Note 3. Alpha-Chymotrypsin modification.

The peptidase aCt was first reacted with azide-NHS ester in order to obtain azide-exposing enzymes to directly conjugate to the alkyne-exposing DV. In order to determine the yield of the first reaction, azide-modified aCt was reacted with an alkyne-modified DNA strand (Supplementary Fig. 11a). SDS-PAGE analysis of aCt, azide-modified aCt (aCt-N3), and DNA-modified aCt (aCt-DNA)

shows that about 50% of aCt-DNA did not react with alkyne-modified DNA (Supplementary Fig. 11b). Considering the high efficiency that usually characterize “click” reactions, this is likely due to a non-complete modification of aCt with azide-NHS ester in the first place. Afterwards we measured the retained enzymatic activity of aCt-N3 and aCt-DNA in DV activity buffer (Buffer 1: TAE + 30 mM $MgCl_2$ + 3 mM $CaCl_2$), and in aCt storage buffer (Buffer 2: 1 mM Tris + 2 mM $CaCl_2$) using the chromogenic substrate sAAPFpNA (Supplementary Fig. 11c).

The product turnover speed of aCt was reduced to 42% after conjugation with NHS ester-azide molecules (Supplementary Fig. 11d). This could indicate that the portion of aCt that was successfully modified (about 50%, as noted above) completely lost activity due to the presence of azide handles. However, after reaction with alkyne-modified DNA the product turnover speed of the enzyme was further reduced to 22% suggesting that a different mechanism could cause the enzymatic activity loss. Finally, proteolytic activity was successfully detected on aCt-loaded DV (Fig. 3e,f and Supplementary Fig. 14) using the fluorogenic substrate FITC-casein, clearly indicating that aCt retained enzymatic activity after conjugation with the DNA origami structure. We thus reasonably ascribe the activity loss measured on chemically-modified enzymes to both the presence of chemical groups on the enzyme surface, and to the enzyme handling along the conjugation procedure in presence of DMSO, copper and chelating agents, and shaking for 3 hours in total at 21 and 25°C (see Materials and methods).

Supplementary Note 4. DNA Vault purification.

The DV was purified by PEG precipitation[1] to remove unconjugated enzymes. The purification efficiency was estimated by testing unmodified aCt in the absence of DNA nanostructures, and assaying both the pellet and supernatant for enzymatic activity by using sAAPFpNA. As expected, aCt was almost exclusively detected in the supernatant (Supplementary Fig. 13a) while the DV is

recovered in the pellet. In order to understand if non-loaded aCt was co-purified with the DV due to unspecific enzyme-DNA interactions the purification protocol was applied on a sample composed of azide-modified aCt and DV lacking the alkyne handle necessary for enzyme loading. Only minor unconjugated enzyme impurities were retained upon purification (Supplementary Fig. 13b).

Supplementary Note 5. Characterization of cargo anchoring site (CAS) region.

As described in the main text, open DV-ssAlk nanostructures were loaded with azide-exposing aCt, purified from excess enzyme, and closed. The sample was then incubated with either OpK or unspecific key. Finally, the chromogenic substrate FITC-casein was added to the samples and the fluorescent signal was measured at specific time-points. Empty open DV was used as control for non-specific degradation of substrate. It was observed that the enzymatic activity measured on open aCt-loaded DV was up to 3 times higher than on closed nanostructures and increasing linearly during the time analysed (Fig. 3e,f).

In order to determine the effect of a stiffer CAS on the overall performance of the DV we performed the same analysis on DV structures assembled with alkyne-modified double-stranded CAS region (DV-dsAlk, Supplementary Fig. 14; Supplementary Table 1). As expected the assay shows similar enzymatic activity values for both open structures. However, proteolytic activity levels measured on closed DV-dsAlk are higher than the ones measured on the DV-ssAlk counterpart. This effect can be ascribed to the presence of a higher amount of non-closed DNA nanostructures, probably because the bulkier and less flexible double-stranded anchoring site used here (about 5 nm long) interferes with the correct closing mechanism of the DV. We have thus identified an effect of the nature of the cargo-anchoring complex on the performance of the DV, indicating that smaller and more flexible anchoring sites allow tighter control over encapsulated enzymes.

Supplementary Note 6. Protocol for loading aCt enzyme in the DNA Vault.

Here we provide a protocol to follow in order to obtain an aCt-loaded DV sample, as the one used in Fig. 3d-f. This protocol can be applied to more different enzymes, but relevant parameters must be changed depending on the nature and chemo-physical properties of the chosen enzyme (e.g. number of lysine residues exposed on the molecular surface, tolerance to temperature, shaking, chemicals, etc.). Also, different enzyme-DNA conjugation strategies may be used to load different enzymes[2], again depending on the nature of the chosen enzyme.

1. <u>Self-assembly of DV-ssAlk in the open conformation.</u> Mix scaffold : staple strands : OpK = 1 : 5 : 50 molar ratio (5 pmol total). TAE/Mg^{2+} buffer (40 mM Tris/Acetate, 1 mM EDTA, pH 8.3, 16 mM $MgCl_2$) is used as folding buffer. Incubate the samples at 75°C for 15 min, followed by temperature ramp of -0.1°C/1.5 minute to 60°C, and afterward a ramp of -0.1°C/6 minutes to 20°C (approximately 39 hours in total).
2. <u>Purification from excess staple strands through PEG-precipitation.</u> Follow the purification protocol and resuspend the pellet in 100 µl DV activity buffer (TAE + 30 mM $MgCl_2$ + 3 mM $CaCl_2$). Add OpK at lock : OpK = 1 : 1.5 molar ratio and incubate the sample at room temperature overnight to allow complete resuspension of the DV.
3. <u>Modification of aCt with NHS ester-azide molecules.</u> aCt (2 nmol) is reacted with NHS-C3-azide linker in lysine : linker = 1:10 molar ratios, and shaken (400 rpm) at 21°C for 2 h in 50 µl final volume of aCt storage buffer. Dilute and wash the sample 2 times with aCt storage buffer by using Amicon Ultra-0.5 ml centrifugal filters (50 kDa) to discard unreacted NHS ester-azide and eventual organic solvents. Purified aCt-N3 may be reacted with alkyne-

modified DNA and analysed by SDS-PAGE, as previously described to estimate the yield of modified enzyme molecules.

4. Conjugation of DV with aCt-N3. React 5 pmol open DV-ssAlk with 10 fold molar excess of purified aCt-N3 by using Oligo-Click-S-Basic reaction kit (baseclick GmbH). 2.5 µl THPTA (100 mM) are added to each reaction tube. The final mix volume should be divided in multiple reaction tubes in order to have about 20-35 µl reaction volume in each tube. Incubate by shaking (500 rpm) at 25°C for 1 h.
5. Purification from excess enzyme through PEG-precipitation. To precipitate the enzyme-loaded DV was mixed with PEG solution and centrifuge at 13,000 rfc for 30 minutes using a cooled centrifuge (10°C). Then, resuspend the pellet in 100 µl DV activity buffer and add ClK at lock : ClK = 1:1.3 molar ratio. Incubate the sample at room temperature overnight to allow complete resuspension of the DV, and subsequently at 4°C for 36 hours to allow full closure of the DV.
6. Closed aCt-encapsulating DV is obtained. The sample may be incubated with either OpK or unspecific key at room temperature for 1 hour before the enzymatic activity assay is performed.

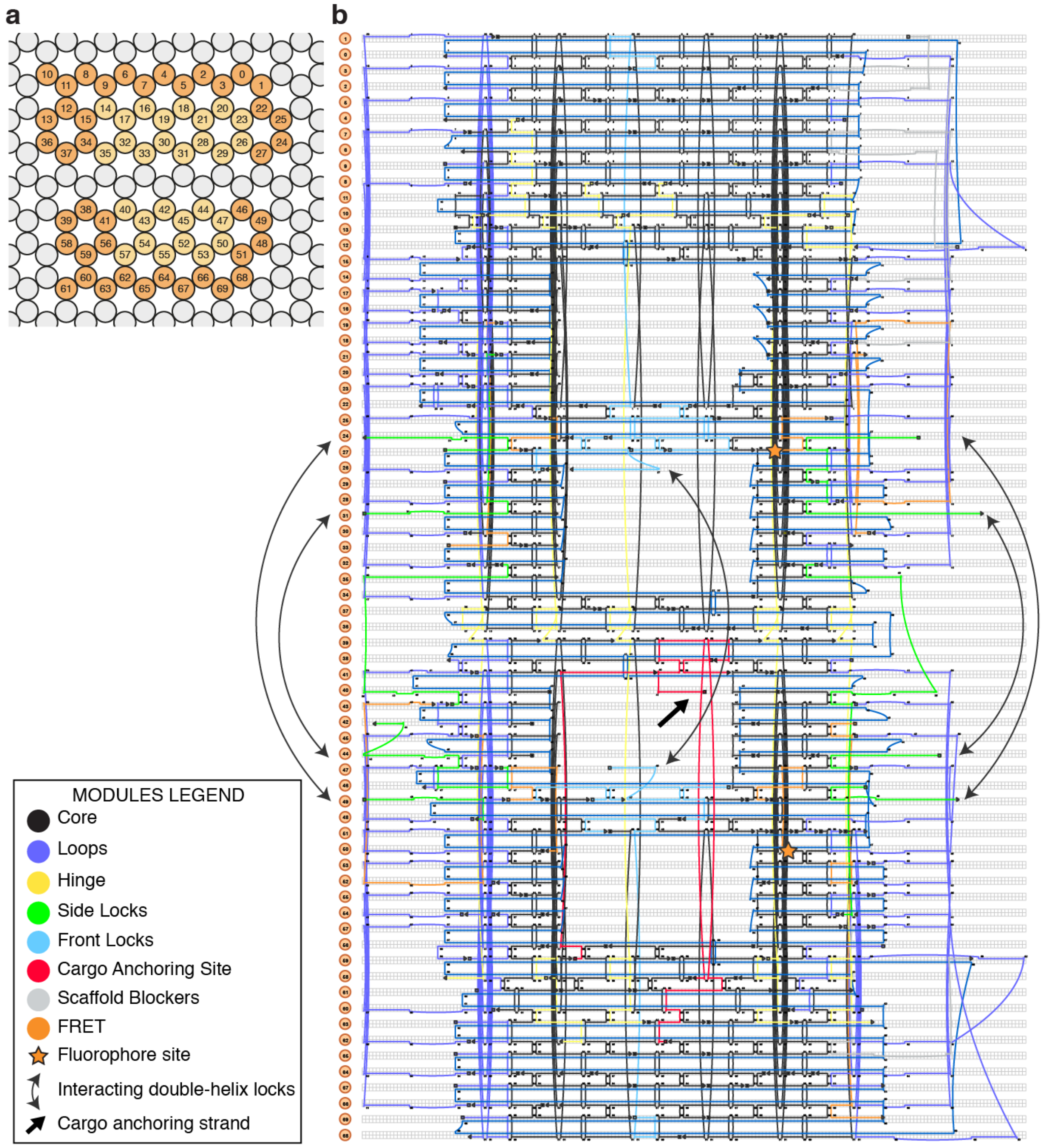

Supplementary Figure 1. DNA Vault blueprint.
Side-view (**a**) and blueprint (**b**), modified from CaDNAno software interface of DV-H1-7K-Cy3/5 as in Supplementary Table 1. Staple strands belonging to different modules of the structure are coloured according to Supplementary Tables 1 and 2, and to the legend in the bottom left corner. The circular DNA scaffold is coloured in blue. Orange stars indicate where Cy3 and Cy5 fluorophores have been positioned for FRET analysis. Curved double-arrows indicate interacting lock strands. The bold arrow indicates the cargo anchoring site (CAS) staple strand.

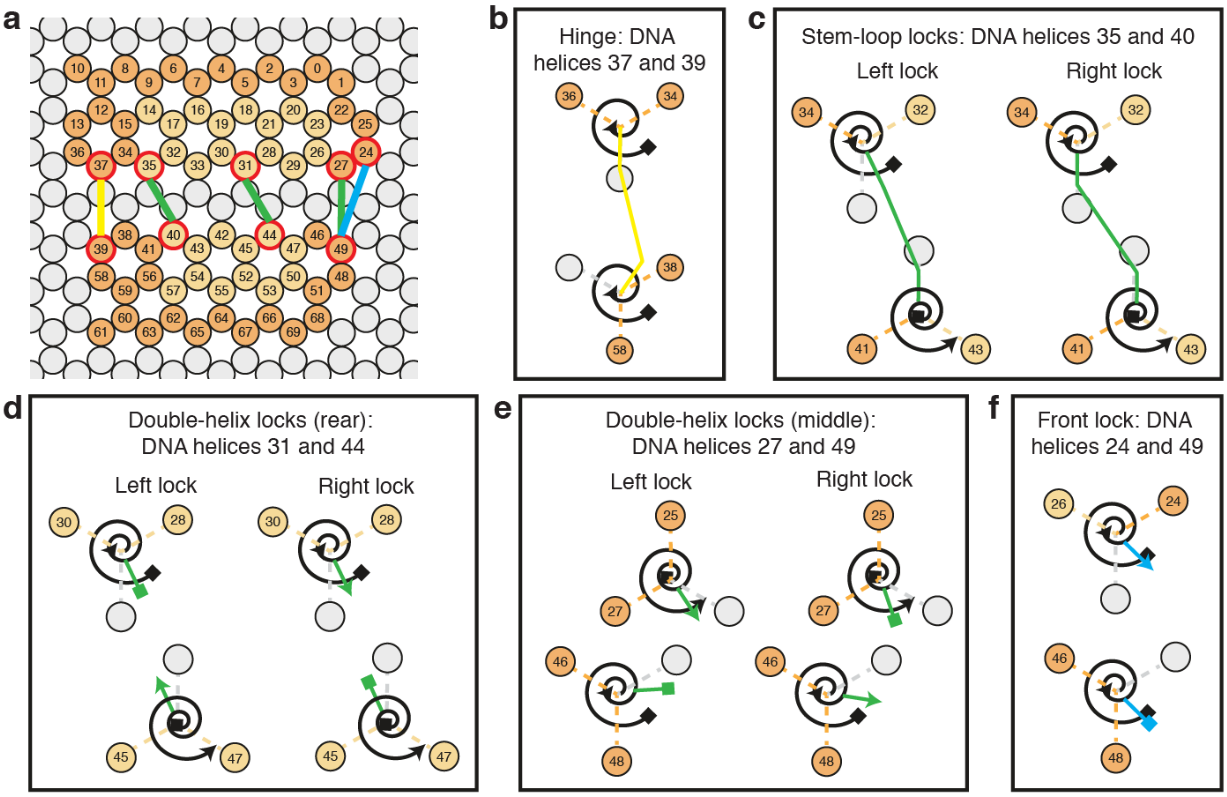

Supplementary Figure 2. Design of the lock system for the DNA Vault.
a, Side-view of the DV highlighting the DNA helices involved in the hinge and lock system design. The yellow, green, and cyan lines link the helices connected by hinge, side locks, and front lock, respectively**. b,** Schematics of the hinge staple strand, connecting helices 37 and 39**. c,** Schematics of the stem-loop locks, connecting helices 35 and 40. **d,** Schematics of the rear double-helix side locks staple strand, protruding from helices 31 and 44**. e,** Schematics of the middle double-helix side locks, protruding from helices 27 and 49. **f,** Schematics of the front double-helix lock, protruding from helices 24 and 49. Dark orange circles indicate DNA helices that form the DV shell, light orange circles indicate DNA helices that define the DV side-walls, grey circles indicate virtual DNA helices used for design purposes only. Spirals show orientation and direction of the DNA helices bearing lock strands (only the staple strand helix is shown for clarity), while yellow, green and cyan lines indicate the hinge and lock staple strand orientations, respectively, on the honeycomb lattice plane as in Supplementary Fig. 1. Arrowheads and squares-on-ends on spirals indicate 3' and 5' ends, respectively.

a

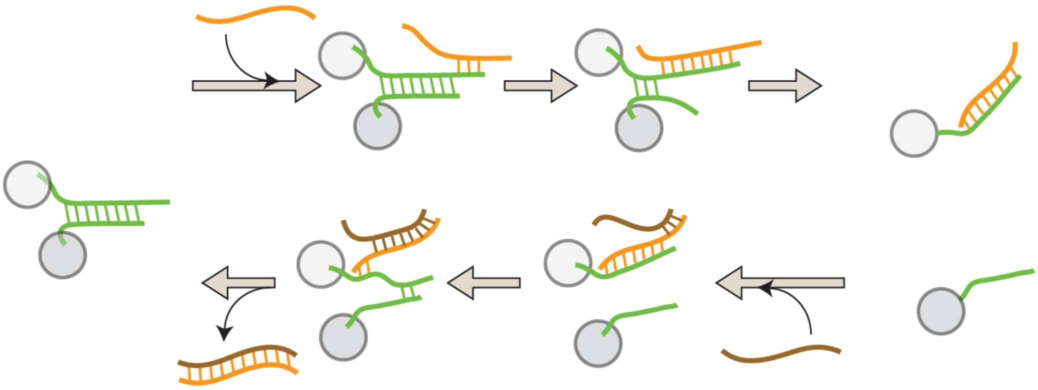

b

```
SIMPLE LOCK STRAND–DISPLACEMENT MECHANISM

Closed Jewel Case
Long lock strand:          JC(5’)–AAAA–BBB–CC–DDDDD–EEEEEEEE–(3’)
                                  ||||  |||  ||  |||||
Short lock strand:         JC(3’)–aaaa–bbb–cc–ddddd–(5’)


The Opening Key is added: open Jewel Case
Long lock strand:          JC(5’)–AAAA–BBB–CC–DDDDD–EEEEEEEE–(3’)
                                       |||  ||  |||||  ||||||||
Opening Key:                      (3’)–bbb–cc–ddddd–eeeeeeee–fffffffff–(5’)

+

Short lock strand:         JC(3’)–aaaa–bbb–cc–ddddd–(5’)


The Closing Key is added: re-closed Jewel Case
Opening Key:                      (3’)–bbb–cc–ddddd–eeeeeeee–fffffffff–(5’)
                                              |||||  ||||||||  |||||||||
Closing Key:                             (5’)–DDDDD–EEEEEEEE–FFFFFFFFF–(3’)

+

Long lock strand:          JC(5’)–AAAA–BBB–CC–DDDDD–EEEEEEEE–(3’)
                                  ||||  |||  ||  |||||
Short lock strand:         JC(3’)–aaaa–bbb–cc–ddddd–(5’)



Notes
A–region is 4–6 nts long
E–region is 8–9 nts long
```

Supplementary Figure 3. Strand displacement mechanism of double-helix locks.
a, Schematic of a double-helix lock connecting the two halves of the DV. The opening key (orange) first anneals onto the toehold exposed on the long lock strand, and then disrupts the lock-to-lock interactions by strand displacement mechanism, causing the structure to open up. The closing key (brown) works similarly: it first anneals onto the toehold exposed on the opening key, and then it disrupts the opening key-to-lock interactions leaving the long lock strand free to interact with the short lock strand, thus inducing the DV to close back. **b,** Sequence domain design of the lock and key strand interactions. Sequence domains are indicated by capital letters and small letters for complementary sequences. Furthermore, the domains are coloured to reference the DNA sequences shown in Supplementary Table 2 (Side-lock and Front-lock modules).

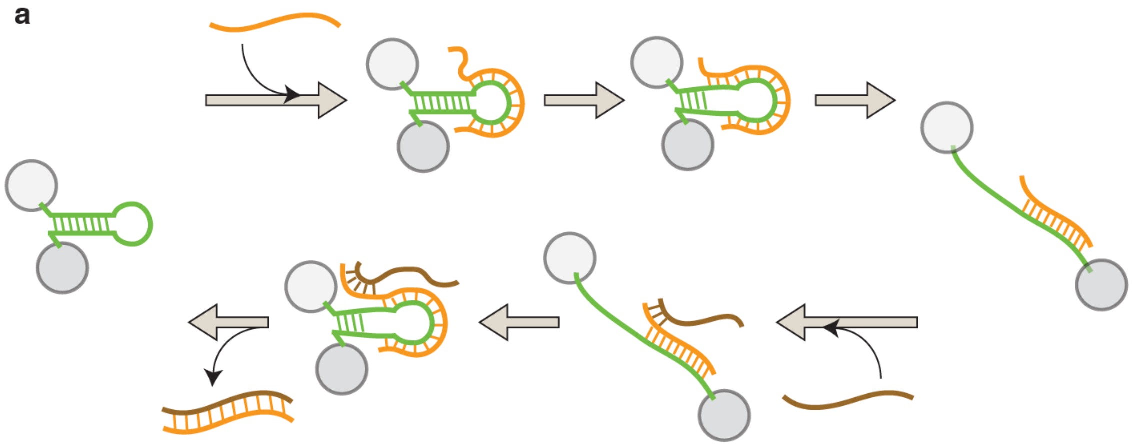

b

```
STEM-LOOP LOCK STRAND-DISPLACEMENT MECHANISM

Closed Jewel Case
Lock strand:          JC(5')-AA-BBB-CC-DDD-EEEE┐
                             ||  |||  ||  |||    |
                      JC(3')-aa-bbb-cc-ddd—EEE┘


The Opening Key is added: open Jewel Case
Lock strand:          JC(5')-AA-BBB-CC-DDD-EEEEEEE-ddd-cc-bbb-aa-----JC(3')
                                |||  ||  |||  |||||||  |||
Opening Key:               (3')-bbb-cc-ddd-eeeeeee-DDD-fffffffff-(5')

The Closing Key is added: re-closed Jewel Case
Opening Key:               (3')-bbb-cc-ddd-eeeeeee-DDD-fffffffff-(5')
                                    ||  |||  |||||||  |||  |||||||||
Closing Key:                   (5')-CC-DDD-EEEEEEE-ddd-FFFFFFFFF-(3')

+

Lock strand:          JC(5')-AA-BBB-CC-DDD-EEEE┐
                             ||  |||  ||  |||    |
                      JC(3')-aa-bbb-cc-ddd—EEE┘
```

Supplementary Figure 4. Strand displacement mechanism of stem-loop locks.
a, Schematic of a stem-loop lock connecting the two halves of the DV. The opening key (orange) first anneals onto the loop toehold exposed on the lock strand, and then disrupts the intermolecular lock-to-lock interactions by the strand displacement mechanism, causing the structure to open up. The closing key (brown) works similarly: it first anneals onto the toehold exposed on the opening key, and then it disrupts the opening key-to-lock interactions leaving the lock strand free to assume the stem-loop secondary structure, thus inducing the DV to close back. **b,** Sequence domain design of the lock and key strand interactions. Sequence domains are indicated by capital letters and small letters for complementary sequences. Furthermore, the domains are coloured to reference the DNA sequences shown in Supplementary Table 2 (Side-lock module).

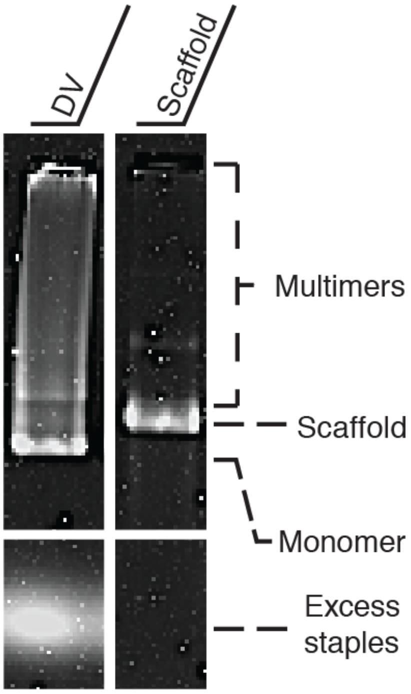

Supplementary Figure 5. Monomer fraction quantification.
Agarose gel analysis of unpurified DV sample. DV monomers constitute roughly 50% of the sample, while dimers and higher-order multimers constitute the remaining 50%.

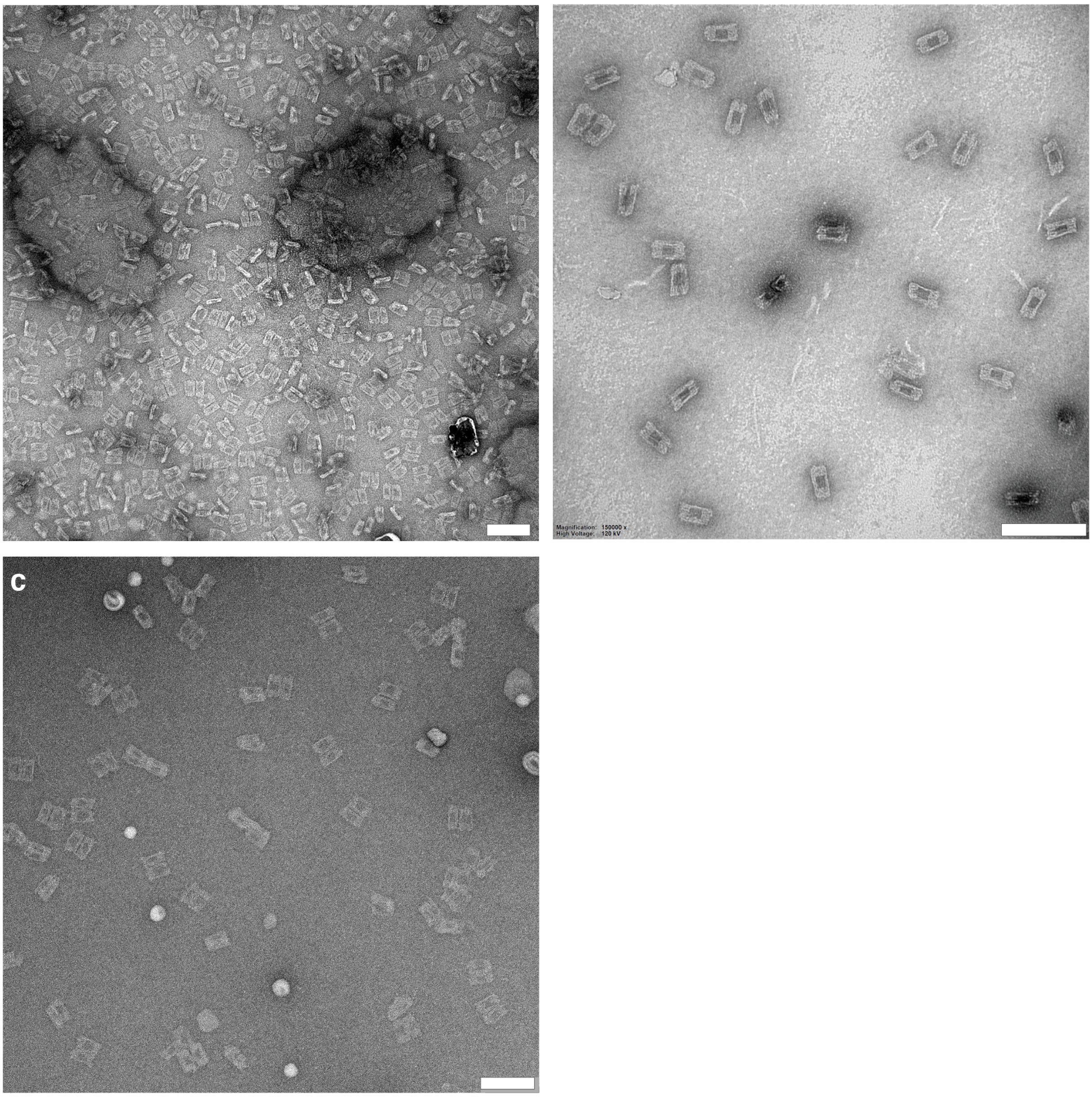

Supplementary Figure 6. TEM images of DNA Vault.
TEM images of the DV (Hinge 3) folded in the open conformation (**a**), after incubation with the closing key (**b**), and subsequently after incubation with the opening key (**c**). Images as seen in Fig. 2b. Scale bar, 100 nm.

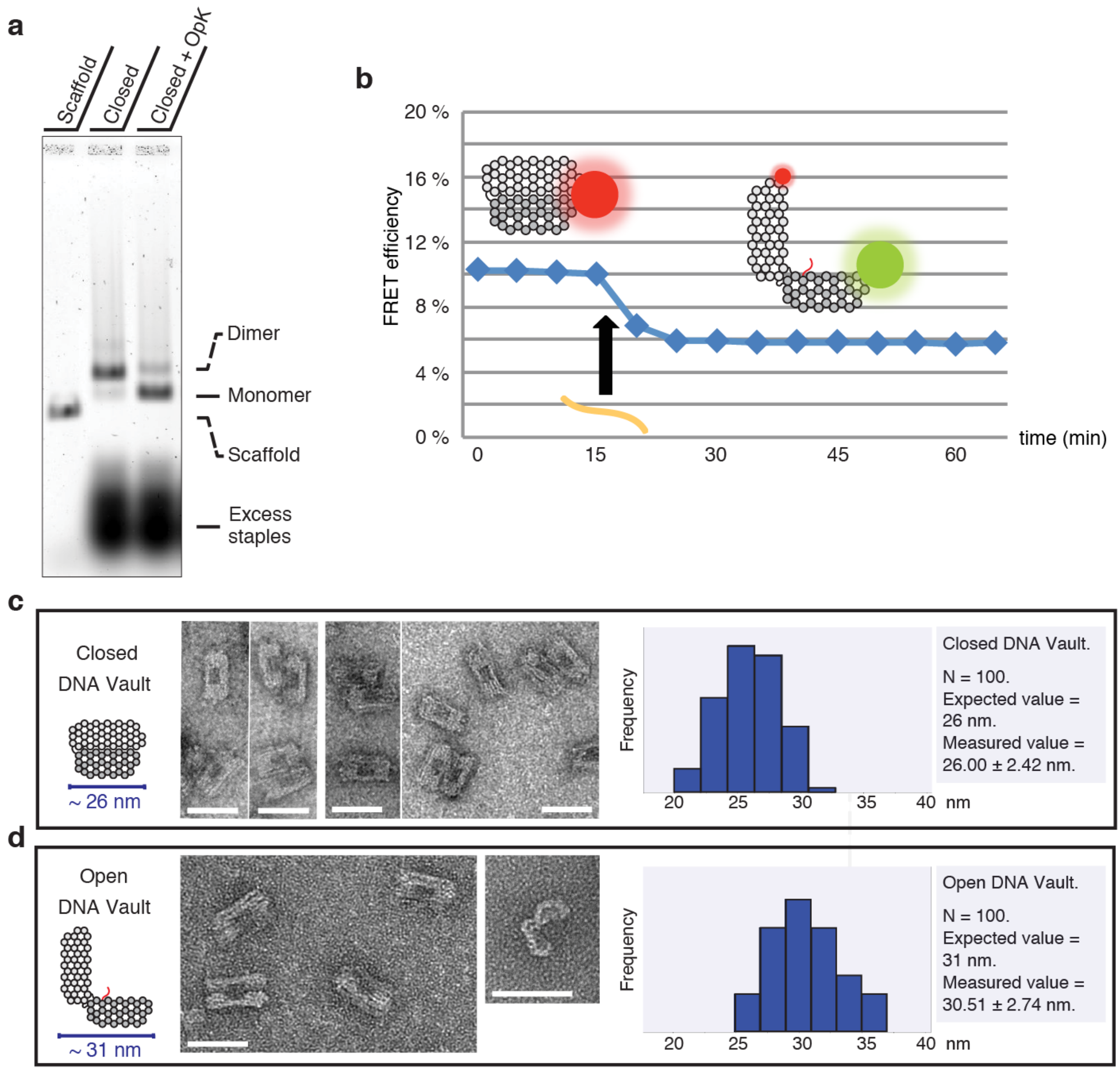

Supplementary Figure 7. Opening mechanism of DNA Vault with stiff hinge (DV-H1).
a, Agarose gel electrophoresis of unpurified DV-H1 assembled in the closed conformation, and after incubation with opening key. **b,** FRET efficiency measurement of closed Cy3/Cy5-modified DV-H1 after incubation with opening key. **c,d,** Schematic (left), and TEM images (middle) of DV-H1 assembled in the closed conformation (c) and after incubation with opening key (d). Dimers are largely present when the DV is assembled closed while almost completely absent after incubation with opening key. The statistical analysis of the back to front length measurements of closed and open structures shows that DV-H1 is opening. Measurements were taken on 100 monodispersed DV-H1 structures. Scale bar, 50 nm.

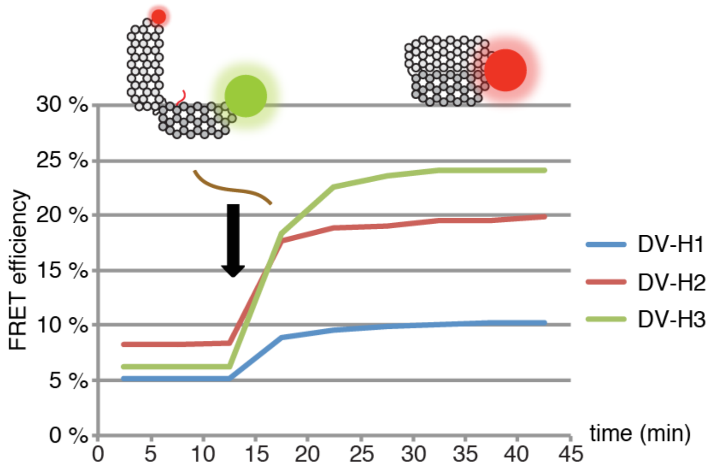

Supplementary Figure 8. Closing mechanism analysis of the DNA Vault with different hinge modules.

FRET efficiency measured on open Cy3/Cy5-modified DV with different hinge modules, after incubation with closing key.

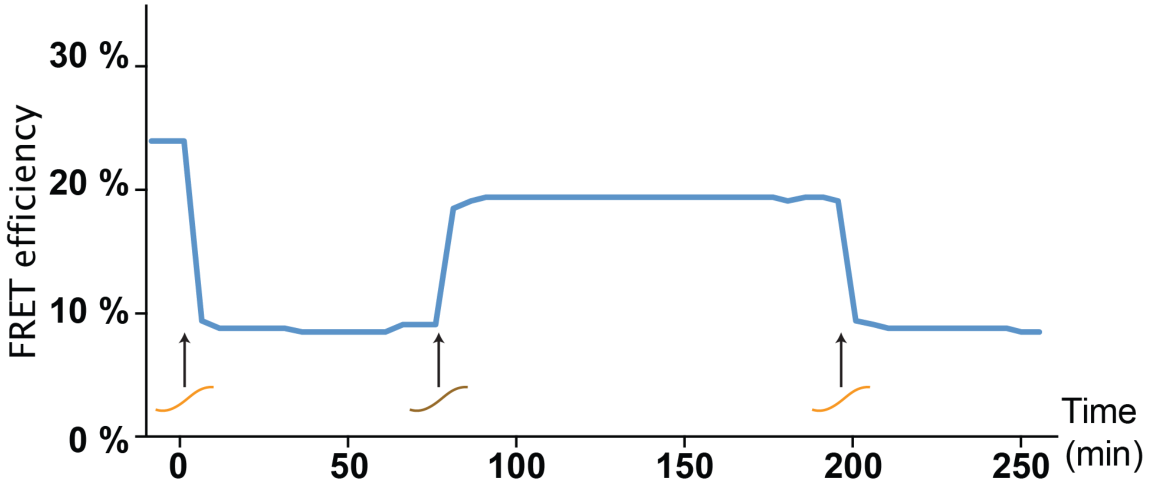

Supplementary Figure 9. Repeated opening and closing of the DV.
FRET measurement of the closed Cy3/Cy5-conjugated DV upon successive addition of opening key (orange), and closing key (brown).

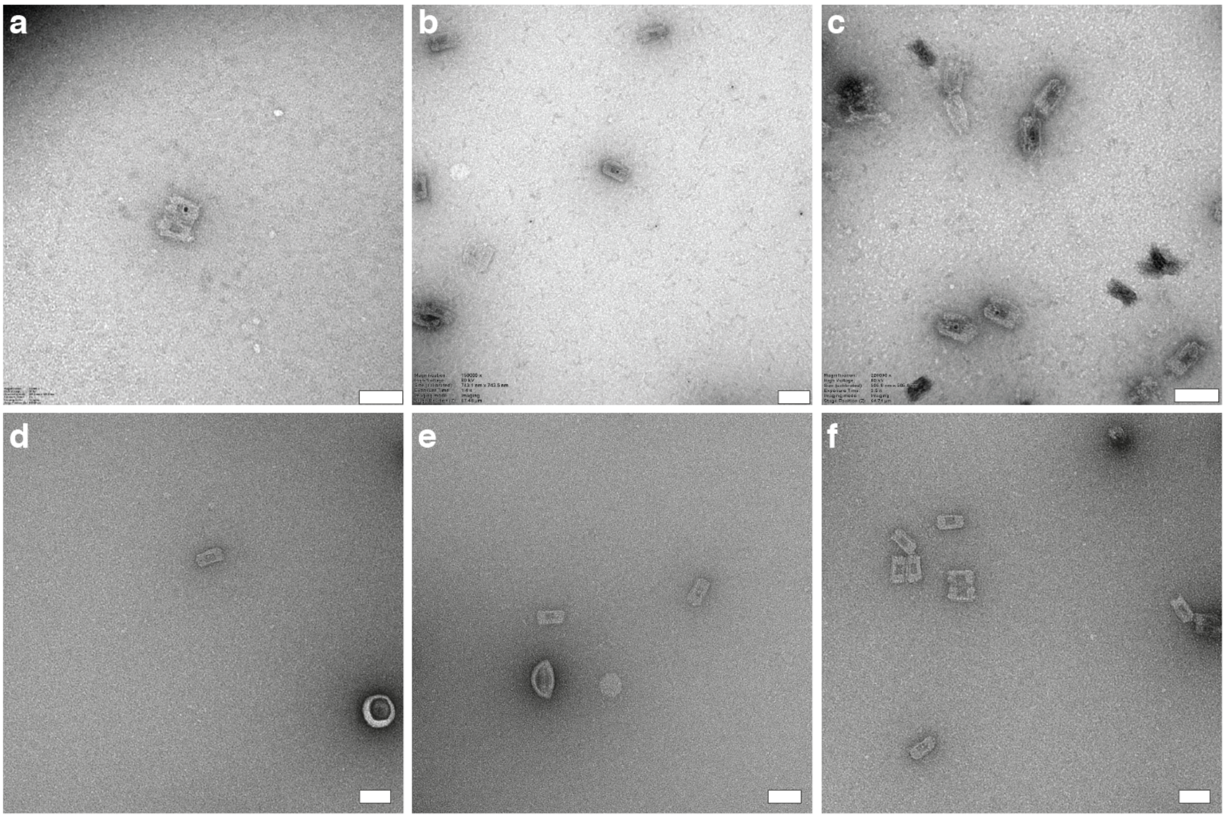

Supplementary Figure 10. TEM images of cargo-loaded DNA Vault.
a-c, TEM images of the DV loaded with AuNPs (5 nm in diameter) previously reacted with thiol-modified DNA. **d-f**, TEM images of the DV loaded with azide-modified alpha-Chymotrypsin. Images as seen in Fig. 3b,d. Scale bar, 50 nm.

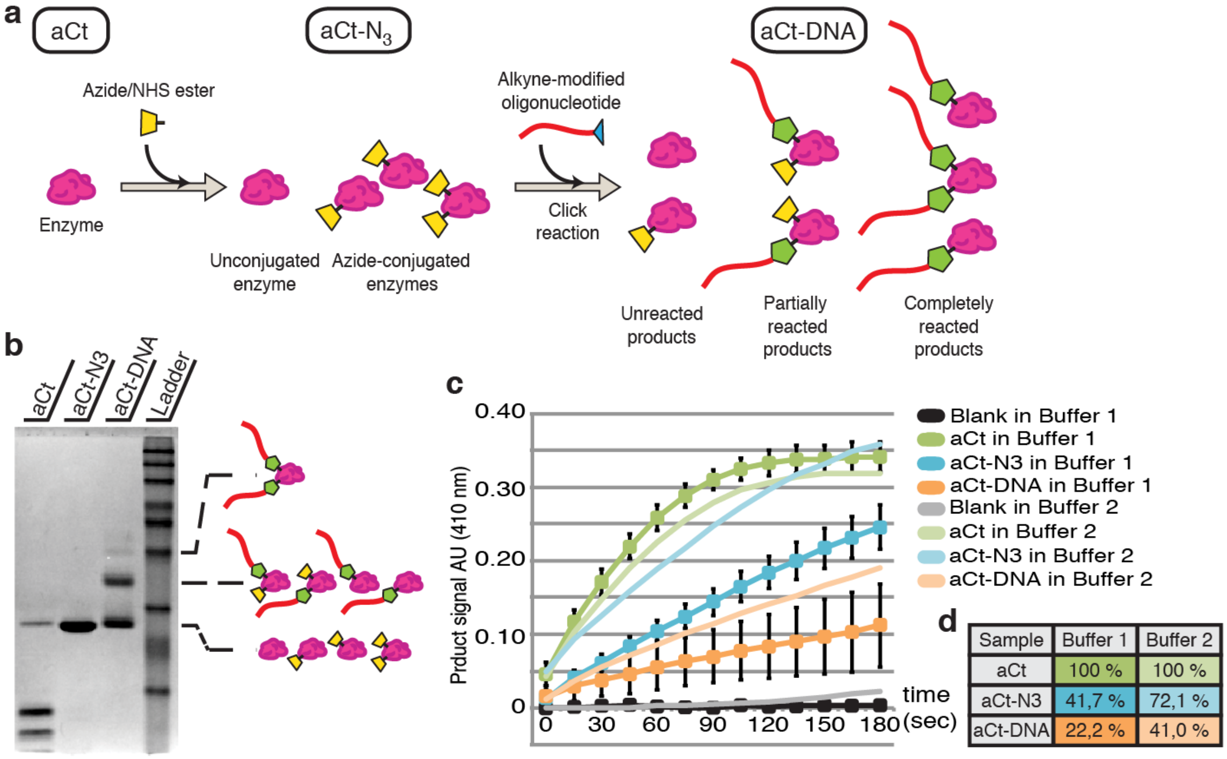

Supplementary Figure 11. Alpha-Chymotrypsin modification.
a, aCt was first reacted with azide-NHS ester in order to obtain azide-exposing enzymes to directly conjugate to alkyne-exposing DV. In order to assess if the enzyme was successfully modified with azide handles prior to covalent loading within the DV, it was reacted with an alkyne-modified DNA strand. **b,** SDS-PAGE of aCt, azide-modified aCt (aCt-N3), and DNA-modified aCt (aCt-DNA). **c,** Retained enzymatic activity was measure on aCt-N3 and aCt-DNA using the chromogenic substrate sAAPFpNA. aCt concentration used was 400 nM, substrate concentration was 400 µM; the tests were performed in DV activity buffer (Buffer 1: TAE + 30 mM $MgCl_2$ + 3 mM $CaCl_2$), and in aCt storage buffer (Buffer 2: 1 mM Tris + 2 mM $CaCl_2$) in triplicates, with error bars indicating one standard deviation. **d,** Quantification of the retained aCt activity as in C.

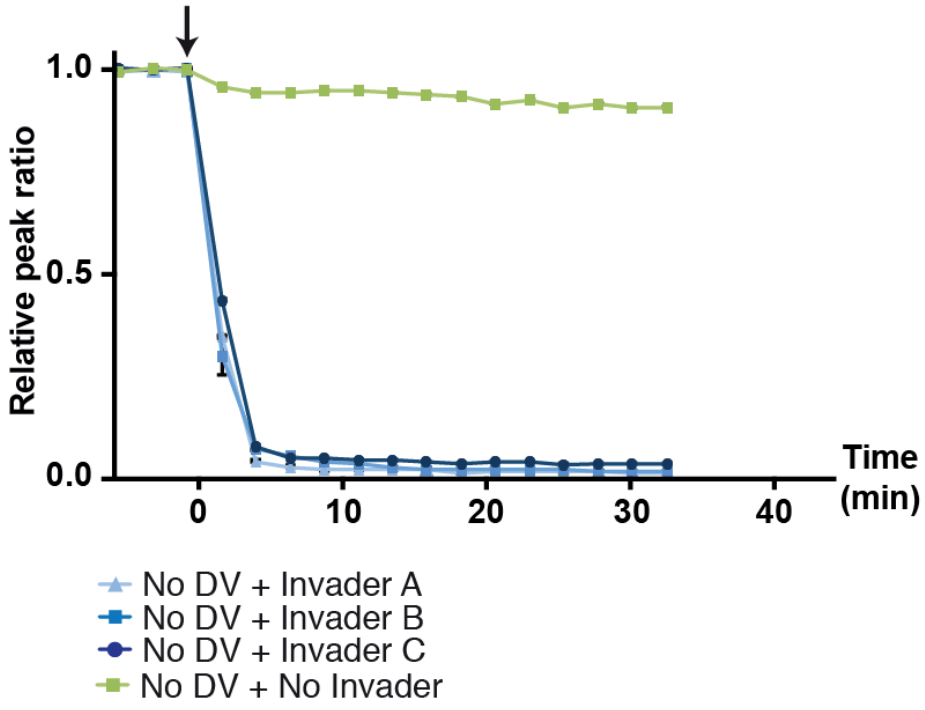

Supplementary Figure 12. Cargo displacement in the absence of the DV.
Cargo displacement reactions were performed on the CAS/cargo system alone in order to determine whether the chemo-physical properties of the different invader species affect the displacement reaction kinetics. Fluorescence data show the decrease of FRET peak intensity (cargo-Cy5 emission upon CAS-Cy3 excitation) upon invader strand addition. Cy3-modified CAS and Cy5-modified cargo (Eco-41[84]-41[104]Cy3 and Cargo-(EcoRV)Cy5, respectively) were pre-incubated together at RT for 30 minutes prior addition of the invader strands. Bars indicate one standard error from the mean fluorescence signal as recorded in three replicates.

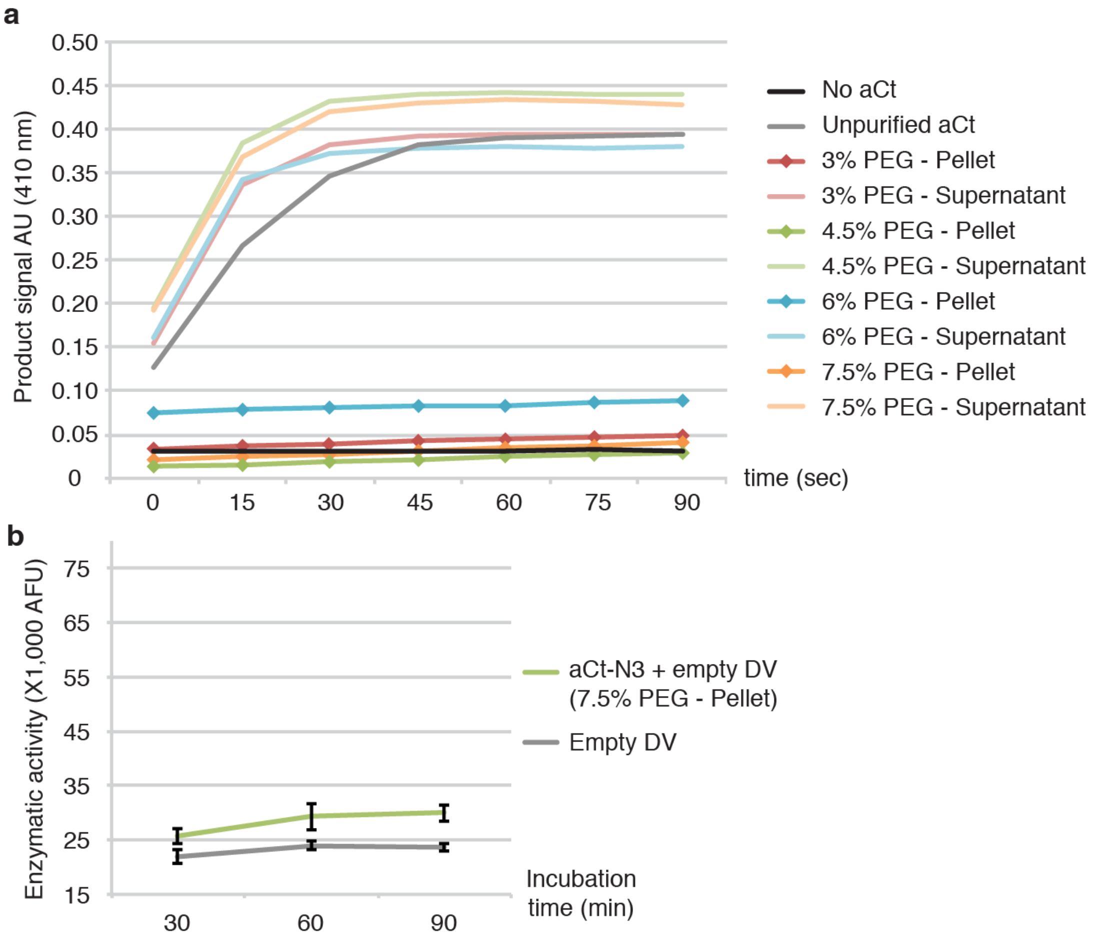

Supplementary Figure 13. Alpha-Chymotrypsin (aCt) activity after PEG-precipitation.
a, In order to determine the purification efficiency of the PEG-precipitation method free unmodified aCt was precipitated using different PEG concentrations, and both pellet and supernatant obtained were tested for retained enzymatic activity using the chromogenic substrate sAAPFpNA. aCt concentration used was 400 nM, substrate concentration was 400 µM; the tests were performed in DV activity buffer (TAE (1X) + $MgCl_2$ (30 mM) + $CaCl_2$ (3 mM)). **b,** In order to understand if non-loaded aCt is co-purified with the DV due to unspecific enzyme-DNA interactions the purification protocol was applied to a sample composed of free azide-exposing aCt and empty DV lacking the alkyne handle necessary for enzyme conjugation. Enzymatic activity was measured on the pellet containing the DV using the fluorogenic substrate FITC-Casein. Enzymatic activity values measured on empty open DV (as in Fig. 4e) are reported here for comparison. Tests were performed in DV activity buffer. Bars indicate one standard error from the mean fluorescence signal as recorded in three replicates.

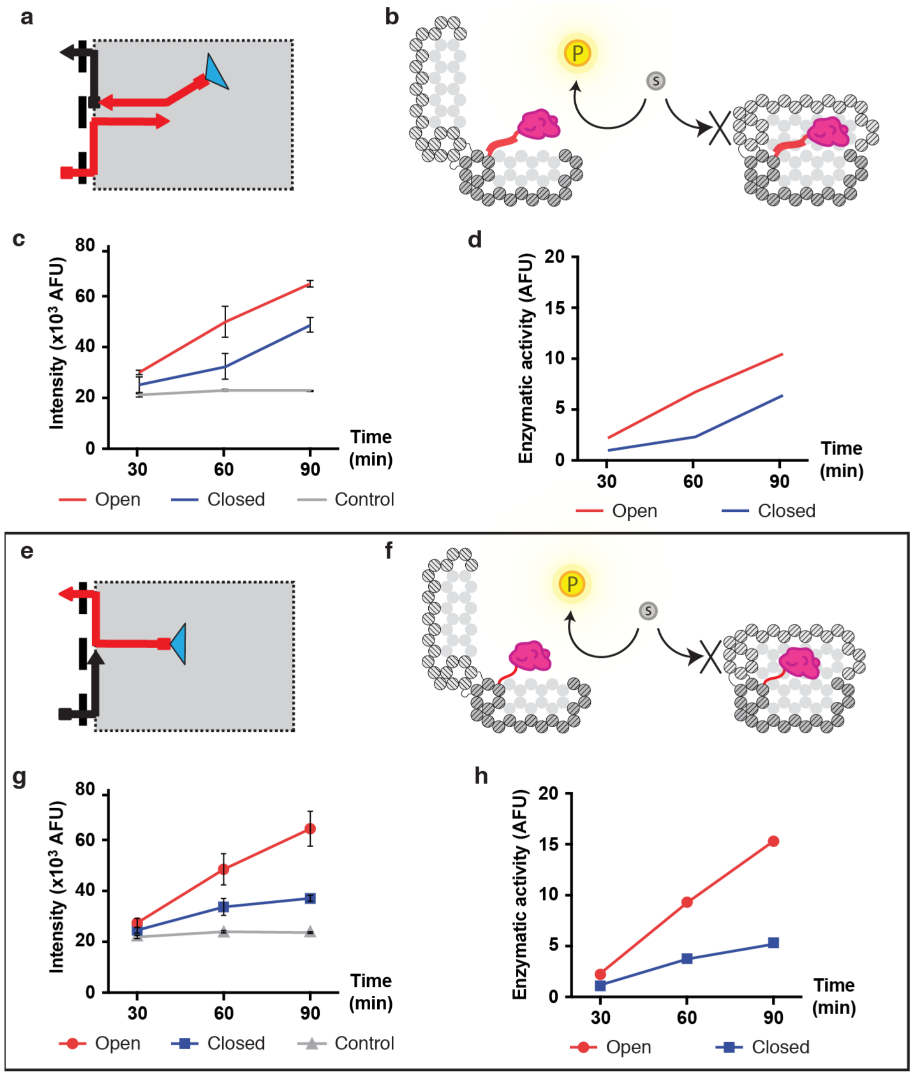

Supplementary Figure 14. DV-aCt enzymatic activity with different protein-anchoring modules.

Alkyne-exposing DV was assembled with either a double-stranded CAS module (DV-dsAlk, A-D) or a single-stranded CAS module (DV-ssAlk, E-H). **a,** Schematics of the protein-anchoring region (the cavity is shown in grey), and **b,** schematic mechanism of the enzymatic activity modulation system in DV-dsAlk. **c,** aCt enzymatic activity measured on open aCt-loaded (blue), closed aCt-loaded (red) and open empty DV-dsAlk (Control, grey). Bars indicate one standard error from the mean fluorescence signal, as recorded in three replicates. **d,** Normalization of measured enzymatic activity as in C after Control subtraction (closed – control at 30 min = 1.0). **e,** Schematics of the protein-anchoring region, and **f,** schematic mechanism of the enzymatic activity modulation system in DV-ssAlk. **g,h,** aCt enzymatic activity measured on DV-ssAlk as shown in Fig. 4e,f. The data relative to DV-ssAlk (bottom box) are shown here for a better comparison with the analysis conducted on DV-dsAlk (top box).

Supplementary Table 1. Module assembly instructions for DNA Vault samples.

Overview of the DNA origami structures, the opening keys (OpK), and closing keys (ClK) used in the experiments. The table lists the 9 different versions of the DV that was assembled for the experiments presented in this study. Each structure is composed of 8 different modules of staple strands, as listed in Supplementary Table 2.

Structure		Modules used									
**DNA Vault**	**Figure**	**Core**	**Hinge**	**Loops**	**Front Lock**	**Side Locks**	**Cargo Anchoring Site**	**Scaffold Blockers**	**FRET**	**Opening Key**	**Closing Key**
DV-H3-7K	2b; Supp. Figs. 6, 11b, 13	Core1	Hinge3	Loops1	Front_Lock2	Side_Lock2	CAS1	Blockers1	FRET1	OpK1/7	ClK1/7
DV-H1-1K-AuNP	2e; Supp. Figs. 1, 8a-c	Core1	Hinge1	Loops1	Front_Lock1	Side_Lock1	CAS3	Blockers1	FRET1	OpK1	ClK1
DV-H1-7K-Cy3/5	Supp. Figs. 5, 7	Core1	Hinge1	Loops1	Front_Lock2	Side_Lock2	CAS1	Blockers1	FRET2	OpK1/7	ClK1/7
DV-H2-7K-Cy3/5	Supp. Fig. 5	Core1	Hinge2	Loops1	Front_Lock2	Side_Lock2	CAS1	Blockers1	FRET2	OpK1/7	ClK1/7
DV-H3-7K-Cy3/5	2c; Supp. Figs. 5, 14	Core1	Hinge3	Loops1	Front_Lock2	Side_Lock2	CAS1	Blockers1	FRET2	OpK1/7	ClK1/7
DV-H3-7K-Displ	3b,c	Core1	Hinge3	Loops1	Front_Lock2	Side_Lock2	CAS2	Blockers1	FRET1	OpK1/7	ClK1/7
DV-H3-7K-ssAlk	2g; 3e,f; Supp. Figs. 8d-f, 10g-h	Core1	Hinge3	Loops1	Front_Lock2	Side_Lock2	CAS4	Blockers1	FRET1	OpK1/7	ClK1/7
DV-H3-7K-dsAlk	Supp. Figs. 10c,d	Core1	Hinge3	Loops1	Front_Lock2	Side_Lock2	CAS5	Blockers1	FRET1	OpK1/7	ClK1/7

Supplementary Table 2. Staple strand sequences for the DNA Vault.
List of all DNA strands used in the study showing the module that they belong to. Modules are coloured as in Supplementary Table 1. Sequences are annotated as follows: (1) Locks/OpK/ClK colour code: they anneal to same-colour sequences (see Supplementary Figs 3 and 4 for more details), (2) Small bold black nts: single-stranded moiety, (3) Small bold red nts: they anneal to other small bold red nts within the same module. (4) Bold blue letters: oligonucleotide modification. Modules are separated by bold lines in the table.

Module	Name	Sequence
Core1	5[123]-27[116]	ATTTACCGTTCCAGTAATAGCGGGGGAAGTTGGGTAAATACACTAATCAA
Core1	0[135]-18[129]	CGAGTTAGCG**tt**TACAAATTCTTAATGCCACAGACTTTGGTAGCAACCC
Core1	1[116]-27[111]	CACAAAGCCAAAGCCTGAACTATATGTAAATTGAATTT
Core1	1[38]-4[45]	ATAGCAAGCCCCAGAACCTTTCGGACCTTGAGACCAGAGGCCA
Core1	11[115]-32[122]	GTAATTTCAGCAGGCTCCAGTGAATTGACAAGAACCGGATTCA
Core1	12[120]-14[115]	AGGCAGATAGAAACGCAATAATAAAAGGGCTAAA
Core1	12[65]-8[81]	AAGCTAATTTGCTAACACAATGAAATAGCAATTAA
Core1	13[26]-12[31]	TGAATTAATAAACCCACAAATTT
Core1	13[60]-9[52]	AGCATTAGTTTAAGAGCATGTTAGCAAACGTCGGA
Core1	13[80]-12[66]	AAATGGAATTGCGCCTTAAATC
Core1	15[137]-36[126]	TATACGTAACAAAGCTGAATTGGGTTAGACT
Core1	15[88]-15[107]	TCAAACTAAAAAACAGCCAAAGCTCTTACCGAAAGGAACCGTTGAAAATCTCCAA A
Core1	16[48]-33[55]	ATTAAACAATCATTACCGC**tt**TAACAGTCAAGTTA
Core1	17[115]-30[122]	TTGAAGAGTAAACCAACTTTGAAAGATAA
Core1	18[128]-15[136]	TCACAGGGAGCGCATAAAGTTGCGCGAGGTGGCCTTTAATTG
Core1	18[47]-31[55]	ATCCCATCATTCCAAGAA**tt**AATCGCGAACATCA
Core1	19[115]-28[122]	GCTGAACCGAATTGTGTCGAAATCCTATC
Core1	20[48]-29[55]	CAATAAACTGAACAAGAAA**tt**AAACAAAACATAAA
Core1	21[115]-23[122]	ATCGGCCTGATCACTCATCTTTGACAAAAGAAATAC
Core1	22[104]-2[108]	ACAGTAGACAACGCCTGTAGCCCGGAATTTAGGATGCGT
Core1	22[44]-26[52]	GGCTTCTGTCCAGAC**tt**ATAT
Core1	22[62]-5[66]	ACATGTAACCCATGTACCGTACACCCTCAACATGATAAGTTTGACA
Core1	22[83]-5[87]	TCGCCATGTTTCGTCACCAGTACTCAGGTGAGACTGATACAGAGAC
Core1	23[123]-2[129]	GTACCAGTATAGACAGCCCTCATAGAGGGTTTCAGTACCTGA
Core1	24[58]-22[63]	TCAGTGTGATAAATAAGAACGCCA
Core1	25[101]-20[107]	ATTACTAGAAAACGCTCA**tt**TAAACTCCAT
Core1	28[121]-1[115]	ATCGAACGAGTTCATGAGTTTTGCGATATAAGTATAGCATTC
Core1	3[56]-25[52]	ATTCTGAAGAACCGCCACCCTAATAGGAATTTAGGACCG
Core1	3[98]-22[94]	AGAAGGAAGGTGTATCACCGTACAAACTGGCT
Core1	32[121]-18[115]	TCATACCGATCCGATATTCGGCATATCTTTTCATAATCAATAAATAAGA
Core1	32[38]-4[23]	TCGGTCGTAGGCAAGTACCCAATGATCAGAGCCACCACCCTCAG
Core1	33[46]-13[38]	TACACCTTTTGTCAGATACGTAAACATATTCTTTTGCGGAACAAACCAA

Core1	34[111]-37[87]	AAGGCTTGCCCTATACTTCTGAATAATGCCTG
Core1	36[104]-9[97]	GTATTAATTTATCCGAATAGAAGCCCTTACCCAAAAGAACTGGGTTTAC
Core1	36[125]-12[121]	TTACAAAAACGATTTCAAC
Core1	36[94]-36[105]	CTTTGTTTGGATTGACGAGAAACACACAACTC
Core1	37[35]-6[28]	TTATCATACAGAAAGCGTTTTGCAAATCACGCAAACATTACCATTAGCA
Core1	37[56]-15[87]	TCAGATGTCAAAATCTTGCGGGAGGTTTTGAAGAATAATAATTTTT
Core1	37[67]-13[59]	CAAAAAGTTTGAGTAACCCAG
Core1	37[77]-37[66]	ATATAATGAAGGGTTAGAACCTACCATAATGG
Core1	37[88]-13[79]	ATTGCCCGAACGTTATTACA
Core1	40[118]-39[113]	TAATGCAGATAC**tt**ACAACATAACTGGCACAACTAAT
Core1	40[53]-39[50]	GCCATTAAAAATCGCCTGCTGCTGAAGTTGGCAAA
Core1	41[116]-64[122]	ACAATGCTGTCCTTTAAGTCACGTTGTGAGCGAGTAACTTGA
Core1	42[111]-64[115]	ACCAA**tttt**ATAACGCCAAAATTAGAGACAAATATTCAG
Core1	42[48]-54[52]	ATACGTGCCCTAAAACATC**tt**GAGA
Core1	44[111]-52[115]	ATCGT**tttt**AATAGCGAGAGGCGAA
Core1	44[128]-40[119]	GCGAAAAGAAGTTTACCCGAGGCAATACCACATTCAAC
Core1	44[48]-66[52]	ACGACCAGACCTGAAAGCG**tt**AAAGGGCAAGTTTTCAAA
Core1	48[90]-68[91]	GGTTGCTAAGGGAAGAAAGCGGGGCGCG
Core1	49[109]-51[115]	ACGCAAACTA**tt**CATAAATATTCAATCAAAATAGG
Core1	49[116]-51[111]	TTATAATGCGCCGCTACCGGGCGC
Core1	49[52]-55[45]	ATCCCGTTAGAATCAGAGTTAGAGCGGTGCCGCCAAATCGAAAAACACGT
Core1	5[56]-20[49]	CAGCATTTAACGGGGTCAGTGACCTATT**tt**GACGA
Core1	5[77]-3[76]	GCAGGTCGAGTGTACTGGTAAAAGTATT
Core1	5[88]-7[76]	GATACCACCGGTTTGCCCCGACTTGAGCCATTTGGGAAAGCGACA
Core1	5[98]-3[97]	GATATTCACATGGCTTTTGATCCTCAAG
Core1	50[51]-64[45]	CGATTGACGGTAGCATTAACATCCGCAAAATCCATCAAATGCCGGAAAT
Core1	51[116]-67[108]	GCGGTTTAGCTATATTTCCCTGTAATGCCTGCTGG
Core1	51[123]-44[129]	CAACCGCGCTACCGTTGTAGCAATACTTGCCCCCCTCAGATA
Core1	51[130]-57[136]	AGCGAGAATGACTATTAATTAAGATAATTCGCCAACAGTTTTGATAAGA
Core1	51[137]-64[129]	ACGTTCGCAAATGGTCAGAAGCCTCCCTCATATGAACGACCC
Core1	51[70]-64[66]	CGTGGCGCATCAATTCTACTATCAGAGCAGGCCGGTTTGAGAGTAA
Core1	52[114]-51[122]	AGAAGGTCTTAAATGCAATACTTTTGCGGGAATAACCTCTGG
Core1	54[51]-66[56]	TAGCTCCAACTTTGTTAAGAGGGTAGCTATTAGACAGT
Core1	56[111]-59[87]	AGCTCAACATGTCCCTGAGAGAGTTGCAGCCC
Core1	56[51]-64[56]	CGAAGAATAGGTATCGGAGGAACGCCATCAATTAATAT
Core1	56[69]-61[76]	CTGGTTTTGAGACGTCGTGCCAGCTGTTTCCTGTGTGAA
Core1	58[106]-63[97]	GTTGAACATTTTCCCAGTCACGACCAACTGT
Core1	58[93]-58[107]	CCCACCGCCTGGTTTAAATATGCAATTGC
Core1	59[88]-56[70]	TTCGCTTTCCAGTATCAAAACGACGGCTGATTGCAAGCGGTCCACG
Core1	60[121]-39[134]	TGGTACGGTGGAATATAGGTAGAAAGATTCAATTACCTTCAATAGAT
Core1	61[117]-41[115]	AAGCATATAACTAGAGCCGTATGCGATTTTAAGTATT
Core1	61[56]-56[52]	CATAGCTGCAAGTTGAACTAAAGCATCACCTAACAGTGCAGG
Core1	61[77]-39[71]	ATTGTTCGGGGTTATCTTGGGAAGAACAAATGAAAAATAGGAATTGA

Core1	63[123]-42[112]	CTTGGGATAGTTGCTCCGTCAGGAGGAATTAAGACGACGATAAAA
Core1	63[81]-65[76]	TCACATCTGCCAGTTTGTCGCGTC
Core1	63[98]-65[97]	TGGGAAGGATGGGCGCATCGTCCAGCTT
Core1	64[114]-50[122]	AAAAAACAAGAGAATCGATATTTTTACCCTGACCA
Core1	64[121]-44[112]	TAACGCGTTTGGAAGCCCTTTTGCTCCAATACTGCGGA
Core1	64[128]-61[139]	CGGAACCCGTCCGTAATCGCTATTGGCGAGTAAAGCCTGGG
Core1	64[44]-61[55]	TCGAACCAATCCTCAGGCTTCTGGTCGACCGTAATCATGGT
Core1	64[65]-59[59]	ACGAAATAATAGGGGACCAAAGCGCCAAGCTTGCATGCTCAC
Core1	65[77]-67[76]	TGGCCTTCAAATATTTAAATTGATCTAC
Core1	65[98]-67[97]	TCATCAAAAAACAGGAAGATTCATTGCC
Core1	66[51]-49[64]	TCATAAGCAATAAAGCCATAGTAGGGAAAGCCTTTCCTTGAGAAGTG
Core1	67[109]-61[116]	AGCAGCCCCACATTAAATGGTGTAGGCGATCCAGGGTACGAGCCGG
Core1	67[77]-69[76]	AAAGGCTCAAAAGGGTGAGAAATAAAGC
Core1	67[98]-69[97]	TGAGAGTAGTAATGTGTAGGTCAAAAAC
Core1	7[56]-18[48]	GCACCGTCCACCCTCAGAACCCCGCCGC**tt**AATAAT
Core1	7[77]-5[76]	GAATCAAGAACCGCCTCCCTCGGTTGAG
Core1	7[98]-5[97]	TCAGACTCCGGAACCAGAGCCTGGCCTT
Core1	8[80]-8[87]	GACTCCTTATTCAATCAATAGAAAATTCATATGCAT
Core1	9[108]-36[95]	GACAACGGAATTTTAAATAACATAAAAACAGGGTATTAATC
Core1	9[123]-16[115]	TTCGGAAATTATTCATTGTTTTCAATTC
Core1	9[53]-11[52]	ATA**tt**GCCCAATAGCAAAGCGAACTGCACCCTAACGAGCGGTCAGCCCAA
Core1	9[56]-16[49]	AGTTTATCAGCAAAATCACCAGATAGCA**tt**CGGGT
Core1	9[98]-7[97]	CAGCGCCAATTATCACCGTCATTTAGCG
Core1	69[98]-49[108]	ATTATGATCATTTGAAAGGAGAGGGCGCGTCTGTCCATC
Core1	11[53]-35[55]	TAAGCTATTTCTCCCGATATTTGCGAATATA
Core1	15[108]-11[114]	AAAAGGAGTGACAATCCAAAGAGAGAAAA
Core1	2[107]-9[107]	CATACAAACAAAAATCAGTAGCGCAAAGGTGAAA
Core1	2[128]-10[128]	ATTAAAGCCAGTTTGCCTTTCGGTTATTGACAACCGATAACCGAGGCCGAATA
Core1	30[121]-5[122]	GGGAGGCTTGGCAGCGACCTC
Core1	37[119]-9[122]	AGTAGTACTCATTCAAAAGGAAATTTCTGACA
Core1	5[67]-8[66]	GGAAGAGCCGAATCAGTTTAGAGCTTTGTCAACGC
Core1	55[46]-60[38]	GGAGGTTGAGAATCAAAAAATCCTGGTGGTTATCGGCCAAAATTTCTAGA
Core1	68[90]-62[87]	AGCTGAAGTTGTACAAAGATTATCAGGTGTATAAGCCTGTAGAACC
Hinge1	10[138]-13[132]	AAATGAAAACAAAGTTATTGCTAAACAACTTTTTTGTT
Hinge1	58[52]-62[56]	ATGATTTCTTTCTGCAGGTGCCGGAAACCAGGGACGACA
Hinge1	59[115]-63[122]	AAAGGTAACGCGGTGCGGGCCT
Hinge1	8[86]-10[75]	GATAGCTAGCATTAGACGGGAG
Hinge1	10[127]-59[114]	GCAGCCTTTACAGATAAGACAATTCGCAGAACGAGATTCACATTAACT
Hinge1	10[34]-58[23]	GCGCCTTAGAAACCAAGCGGAAAATATGGGGAGAGGCGG
Hinge1	10[74]-63[80]	AATCAGTTAATTTTATTCATCAGGAAGAAACCTGGGCAACAGCCAGTGCCATTCG CCAT
Hinge1	8[65]-58[53]	AGTAAGAATGAACACCCTGAACAAATCTTTATTATCACTGATTATCAACTTA
Hinge1	13[133]-63[145]	TAACGTCAGAAGTACTTGAGAAATACTGCCTAAGTTTCATGCTGCAAACGCCAGC TGGCG

Hinge2	10[138]-13[132]	AAATGAAAACAAAGTTATTGCTAAACAACTTTTTTGTT
Hinge2	58[52]-62[56]	ATGATTTCTTTCTGCAGGTGCCGGAAACCAGGGACGACA
Hinge2	59[115]-63[122]	AAAGGTAACGCGGTGCGGGCCT
Hinge2	8[86]-10[75]	GATAGCTAGCATTAGACGGGAG
Hinge2	Hinge2_10[127]-59[114]	GCAGCCTTTACAGATAAGACAATTCGCAGAACG**tttt**AGATTCACATTAACT
Hinge2	Hinge2_10[34]-58[23]	GCGCCTTAGAAACCAAGCGGAA**tttt**AATATGGGGAGAGGCGG
Hinge2	Hinge2_10[74]-63[80]	AATCAGTTAATTTTATTCATCA**tttt**GGAAGAAACCTGGGCAACAGCCAGTGCCATTCGCCAT
Hinge2	Hinge2_8[65]-58[53]	AGTAAGAATGAACACCCTGAACAAATCTTTATTATCACTGATTA**tttt**TCAACTTA
Hinge2	Hinge2_13[133]-63[145]	TAACGTCAGAAGTACTTGAGA**tttt**AATACTGCCTAAGTTTCATGCTGCAAACGCCAGCTGGCG
Hinge3	10[138]-13[132]	AAATGAAAACAAAGTTATTGCTAAACAACTTTTTTGTT
Hinge3	58[52]-62[56]	ATGATTTCTTTCTGCAGGTGCCGGAAACCAGGGACGACA
Hinge3	59[115]-63[122]	AAAGGTAACGCGGTGCGGGCCT
Hinge3	8[86]-10[75]	GATAGCTAGCATTAGACGGGAG
Hinge3	Hinge3_10[127]-59[114]	GCAGCCTTTACAGATAAGACAATTCGCAGAACG**tttttttt**AGATTCACATTAACT
Hinge3	Hinge3_10[34]-58[23]	GCGCCTTAGAAACCAAGCGGAA**tttttttt**AATATGGGGAGAGGCGG
Hinge3	Hinge3_10[74]-63[80]	AATCAGTTAATTTTATTCATCA**tttttttt**GGAAGAAACCTGGGCAACAGCCAGTGCCATTCGCCAT
Hinge3	Hinge3_8[65]-58[53]	AGTAAGAATGAACACCCTGAACAAATCTTTATTATCACTGATTA**tttttttt**TCAACTTA
Hinge3	Hinge3_13[133]-63[145]	TAACGTCAGAAGTACTTGAGA**tttttttt**AATACTGCCTAAGTTTCATGCTGCAAACGCCAGCTGGCG
Loops1	61[32]-41[31]	CGAGCTCGCGCGCCTGGTCACCTCAAATATCAAAGTCA
Loops1	32[30]-16[24]	AACAATAA**tttttttttttttttttttttttttttt**AATCAATAATCGATCG
Loops1	22[30]-20[22]	AGTAATAAGATACCGACAATGCA
Loops1	12[30]-17[20]	TATACGCGAGTAAAGAAATTGC**tttttttttttttttttttttttttttt**CAAGC
Loops1	13[39]-8[30]	CGCAGCTACAGAATTGAACATACATAAAG
Loops1	14[139]-30[145]	TTGCTTTCCGACAATGAC**tttttttttttttttttttttttttttt**GAAC
Loops1	15[140]-33[139]	CGGT**tttttttttttttttttttttttttttt**CCAGGCGCATAGGCTGATGAACG
Loops1	16[139]-32[130]	TCGCCCATTAAAGGGCAGACGGTCAATCAGGACAGGCT
Loops1	16[20]-21[30]	ACAAG**tttttttttttttttttttttttttttt**AGCCGCCACCAGTTACGAGACA
Loops1	16[23]-7[29]	AGACGTTTTTGAAGGCTTATCC**tttttttttttttttttttttttttttt**GGAAA
Loops1	18[139]-28[136]	GATCGTCACGGCTAACAA
Loops1	18[22]-23[30]	AGAAACC**tttttttttttttttttttttttttttt**AGTTAATGCCCCTTCAGCTAAA
Loops1	19[39]-2[23]	TTATCCTAATAACCACCTAACAGTGCCCGTATAAAC
Loops1	20[136]-0[136]	CTAATACGAAGGCACC**tttttttttttttttttttttttttttt**TGCCGT
Loops1	20[21]-22[31]	GAACGC**tttttttttttttttttttttttttttt**CCCTCATTTTCAAGCC
Loops1	21[31]-22[16]	ATAGACAATTTCATTT**tttttttttttttttttttttttttttt**TAAAGGAATA
Loops1	21[39]-0[23]	GTCCAACATGCTGCCTAGCCACCCTCAGAGCCACCA
Loops1	25[133]-27[138]	CGTTA**tttttttttttttttttttttttttttt**AACCTAAAACGAGACTAC
Loops1	28[135]-20[137]	CGGGATTATACCAAGCGCG**tttttttttttttttttttttttttttt**TTGAGGA
Loops1	29[23]-1[37]	GAATTACCTTTTTTAATGGATAACCTAAAGTAAATTTTCGGGG
Loops1	30[29]-18[23]	TCAATTA**tttttttttttttttttttttttttttt**GCCTGTTTATCACATGT
Loops1	38[139]-54[140]	ATTGTGATCAGTT**tttttttttttttttttttttttttttt**AGACCGGAA

Loops1	4[137]-16[140]	TTAGCGAATGGAAAtttttttttttttttttttttttttttttACCA
Loops1	41[32]-59[29]	GTAGGTGGTTCCGAAttttTTTGCGT
Loops1	43[140]-52[140]	AGCAtttttttttttttttttttttttttttttGGATTGCAT
Loops1	45[140]-51[136]	CAGAGGGGTtttttttttttttttttttttttttttttAGTTCAGAAAACGGTC
Loops1	47[140]-51[129]	TAAACtttttttttttttttttttttttttttttAACCACCACACCCGGTGT
Loops1	47[32]-43[24]	ATTCTAAATCGGAACtttttttttttttttttttttttttttttCAGATTTGAATCTTT
Loops1	48[31]-46[17]	ACAGGAGGCCGATtttttttttttttttttttttttttttttATCGTCCGCT
Loops1	52[37]-47[31]	TATCCCTTCTGTAATAAAAGGGACGATT
Loops1	53[145]-51[143]	AAGCtttttttttttttttttttttttttttttCATTAGATACATCTGC
Loops1	54[135]-67[148]	ACTAGCTTCACATATGTGTAATCGTAAAACTAG
Loops1	54[28]-38[24]	TGGAACtttttttttttttttttttttttttttttAGGTGAGGCGCCCT
Loops1	54[37]-45[31]	GTTCTGATAGGCACAGACAATATTGATA
Loops1	55[145]-53[144]	AACCtttttttttttttttttttttttttttttATAAAAATTAAGCA
Loops1	56[148]-60[122]	GGATGGCTTAGAGCTTAATTGCTTCTGGAATGAGTGAGCAAGTTTAAGT
Loops1	57[145]-55[144]	TTGCtttttttttttttttttttttttttttttCATGTCAATAAGCG
Loops1	59[30]-62[31]	ATTGGTCCCCGGtttttttttttttttttttttttttttttATCGGCAAAATCCACT
Loops1	60[142]-57[144]	TGTTCCATtttttttttttttttttttttttttttttACAAACGGCCATTT
Loops1	60[37]-54[29]	GGAGCGCCAGGTTTGATTTAACACACCGAACGAACCACGCGCGAACCAGTT
Loops1	9[23]-32[31]	CATATAAAAGAAAGATATAATTTTCAGAGA
Loops1	62[30]-64[31]	CCAGCtttttttttttttttttttttttttttttAAGAGTCCACTATTTT
Loops1	63[26]-54[38]	CAGCTTTCCGGCACCGAAGATCGCCTTATATGTT
Loops1	64[30]-66[31]	TGTTAtttttttttttttttttttttttttttttCCACTACGTGAATTCA
Loops1	65[26]-52[38]	AATCAGCTCATTTTTTCATTAAATTAAAGACGTC
Loops1	66[30]-68[30]	ACCGTtttttttttttttttttttttttttttttCCTAAAGGGAGCCATAC
Loops1	67[26]-47[38]	TCTAGCTGATAAATTATATGATACCATCACTAAAGCATACA
Loops1	69[28]-49[40]	AAGGCAAAGAATTAAATAAATCCCCGATCGGGAGCAGGAAC
Loops1	8[136]-12[140]	AGGATGAGGGAGGtttttttttttttttttttttttttttttGGATT
Loops1	8[29]-15[27]	GTGGCAAtttttttttttttttttttttttttttttGGTATTCTAAGA
Loops1	7[30]-30[30]	CGTCACGCACTCGCTGTCTTCATT
Loops1	23[31]-22[45]	AGGTTGCTTCTGTAAAtttttttttttttttttttttttttttttAAATTTAATGGTTTGAAATCAGA
Front_Lock1	22[93]-27[86]	TAACGGAATCCAAATCCAATCGCAGAC
Front_Lock1	Set1-24[69]-26[59]	AGAAAACCATAGCGATAGCTttttTGGTATAAACATCAGTGTAGGAT
Front_Lock1	26[51]-24[59]	ATGAGAATCCTTGAAAATTTT
Front_Lock1	27[76]-22[84]	TAGATTAAAGACAAAATAAGAATAAACACTTGAGAA
Front_Lock1	27[87]-25[100]	GCTGAGAAGAGTCAATAGGCTGATGATA
Front_Lock1	3[77]-24[70]	AAGAGGCAGGTTTAGTACCGCACACTGAATTTAACGCGTTAAGAACGCG
Front_Lock1	46[104]-48[91]	TCGGCCTTGCTGGTTAAAAGAGTACTAT
Front_Lock1	Set1-47[67]-49[74]	TGATGTTTATACCACAGTGAGGCCACCGAGAATATCCAGAACAATATTACCATAAT
Front_Lock1	49[65]-49[51]	TTTTTGCCAGCCATTGCAACAGGAAAAGA
Front_Lock1	69[77]-51[69]	TAAATCGAAGGTGGAGAAAGGTTGACGAGCACGTATAACGTGCGGCGAA
Front_Lock1	27[46]-33[45]	CTTTGAGTGAAAACAGTATTAATTTGAAACACAGAGGCTGAA

Front_Lock2	22[93]-27[86]	TAACGGAATCCAAATCCAATCGCAGAC
Front_Lock2	Set4-24[69]-26[59]	AGAAAACCATAGCGATAGCTttttTCAACATCAGTCTGATAAGCTA
Front_Lock2	26[51]-24[59]	ATGAGAATCCTTGAAAATTTT
Front_Lock2	27[76]-22[84]	TAGATTAAAGACAAAATAAGAATAAACACTTGAGAA
Front_Lock2	27[87]-25[100]	GCTGAGAAGAGTCAATAGGCTGATGATA
Front_Lock2	3[77]-24[70]	AAGAGGCAGGTTTAGTACCGCACACTGAATTTAACGCGTTAAGAACGCG
Front_Lock2	46[104]-48[91]	TCGGCCTTGCTGGTTAAAAGAGTACTAT
Front_Lock2	Set4-47[70]-49[74]	CAGACTGATGTTGACAGTGAGGCCACCGAGAATATCCAGAACAATATTACCATAAT
Front_Lock2	49[65]-49[51]	TTTTTGCCAGCCATTGCAACAGGAAAAGA
Front_Lock2	69[77]-51[69]	TAAATCGAAGGTGGAGAAAGGTTGACGAGCACGTATAACGTGCGGCGAA
Front_Lock2	27[46]-33[45]	CTTTGAGTGAAAACAGTATTAATTTGAAACACAGAGGCTGAA
Side_Lock1	43[25]-32[39]	AATCAGCAGAAGATAAATGTAAACATCAGTGTAGGATGTTTACAtttttGTAGATTTTCAGGTTTAACACA
Side_Lock1	Set1-47[39]-44[0]	TTGATGGAAATACCTACATTTTGATGAAATGATTCTttttggtaTAAACATCAGTGTAGGAT
Side_Lock1	Set1-31[0]-21[38]	TGATGTTTAtaccaCCTGAGCAAAAGAAGATGAACATTTAATAA
Side_Lock1	Set1-27[23]-24[0]	TCGTCGCTATTAATTAATTAATTTCATCTTCTGtttggtaTAAACATCAGTGTAGGAT
Side_Lock1	Set1-49[0]-48[32]	TGATGTTTAtaccaAAGGGATTTTAGACTAA
Side_Lock1	Set1-46[143]-49[167]	GTAATAACATCACTTCtttggtaTAAACATCAGTGTAGGAT
Side_Lock1	Set1-24[167]-29[128]	TGATGTTTAtaccaAACCTCCGGCTTAGGTTGGTCTGAGAAAGAGGCCCC
Side_Lock1	Set1-29[129]-31[167]	CAGCAGATTTGGCGACCTGCTCCATGTTACTTAGCttttggtaTAAACATCAGTGTAGGAT
Side_Lock1	Set1-44[167]-47[139]	TGATGTTTAtaccaGTAAAATGTTTAGACTGAATGCTT
Side_Lock1	32[129]-54[136]	GACCTATTCATTACCCAAttttTGTAAACATCAGTGTAGGATGTTTACATTTAGGATAGTAAGGCAA
Side_Lock2	Set1-43[25]-32[39]	AATCAGCAGAAGATAAATGTAAACATCAGTGTAGGATGTTTACAtttttGTAGATTTTCAGGTTTAACACA
Side_Lock2	Set2-47[39]-42[0]	TTGATGGAAATACCTACATTTTGATGAAATGATTCTttttTGGGGTATTTGACAAACTGACA
Side_Lock2	Set2-31[0]-21[38]	TGTCAAATACCCCACCTGAGCAAAAGAAGATGAACATTTAATAA
Side_Lock2	Set3-27[33]-24[0]	TCGTCGCTATTAATTAATTAATTTCATCTTCTGtttCAGCTGCTTTTGGGATTCCGTTG
Side_Lock2	Set3-49[0]-48[32]	TCCCAAAAGCAGCTGAAGGGATTTTAGACTAA
Side_Lock2	Set5-46[143]-49[170]	GTAATAACATCACTTCttTCAGCCGCTGTCACACGCACAG
Side_Lock2	Set5-24[158]-29[128]	GTGACAGCGGCTGAAACCTCCGGCTTAGGTTGGTCTGAGAAAGAGGCCCC
Side_Lock2	Set6-29[129]-31[176]	CAGCAGATTTGGCGACCTGCTCCATGTTACTTAGCttttACTCACCGACAGCGTTGAATGTT
Side_Lock2	Set6-44[164]-47[139]	ACGCTGTCGGTGAGTGTAAAATGTTTAGACTGAATGCTT
Side_Lock2	Set7-32[129]-54[136]	GACCTATTCATTACCCAAttttTTAATGCTAAATCACGATTAGCATTAATTTAGGATAGTAAGGCAA
CAS1: empty	PAS2_38[99]-41[104]	ATACCAGTCAACGAACTAACGGA
CAS1	PAS2_62[86]-38[100]	GTGGGCTGCGGTTGTCGCTCACAATTCCACACCGCTCAGCACTATCATT
CAS1	PAS3_59[60]-41[83]	CAGGCCCCAGCCACGCTGAGAGCCAGCAGAAATCTACG
CAS1	40[97]-58[94]	ttcgctgccttgtcgTTAATAAAGGACGTAAAATATCTTTAGGACTG
CAS2: Cargo Displacement	PAS2_38[99]-41[104]	ATACCAGTCAACGAACTAACGGA
CAS2	PAS2_62[86]-38[100]	GTGGGCTGCGGTTGTCGCTCACAATTCCACACCGCTCAGCACTATCATT
CAS2	Eco-41[84]-41[104]Cy3	/5Cy3/gaaggatatcaggctctctctcTTAATAAAACGAACTAACGGA

CAS2	PAS3_59[60]-41[83]	CAGGCCCCAGCCACGCTGAGAGCCAGCAGAAATCTACG
CAS2	Cargo-(EcoRV)Cy5	/5Cy5/gcctgatatccttcCTCGTATG
CAS3: AuNPs	38[90]-58[94]	AGGACGTAAAATATCTTTAGGACTG
CAS3	PAS2_38[99]-41[104]	ATACCAGTCAACGAACTAACGGA
CAS3	PAS2_59[60]-40[70]	CAGGCCCCAGCCACGCTGAGAGCCAGCAGAAATCTACGcgacaaggcagcga
CAS3	PAS2_62[86]-38[100]	GTGGGCTGCGGTTGTCGCTCACAATTCCACACCGCTCAGCACTATCATT
CAS3	PAS2_40[97]-(5')SH (to be conjugated on AuNPs)	/5ThioMC6-D/ttcgctgccttgtcgTTAATAA
CAS4: ssAlk	PAS2_38[99]-41[104]	ATACCAGTCAACGAACTAACGGA
CAS4	PAS2_62[86]-38[100]	GTGGGCTGCGGTTGTCGCTCACAATTCCACACCGCTCAGCACTATCATT
CAS4	PAS3_59[60]-41[83]	CAGGCCCCAGCCACGCTGAGAGCCAGCAGAAATCTACG
CAS4	PAS3_40[97]-58[94]-ssAlk	/5Hexynyl/ttcgctgccttgtcgTTAATAAAGGACGTAAAATATCTTTAGGACTG
CAS5: dsAlk	PAS2_38[99]-41[104]	ATACCAGTCAACGAACTAACGGA
CAS5	PAS2_62[86]-38[100]	GTGGGCTGCGGTTGTCGCTCACAATTCCACACCGCTCAGCACTATCATT
CAS5	PAS3_41[84]-58[94]_cut	TTAATAAAGGACGTAAAATATCTTTAGGACTG
CAS5	PAS2_59[60]-40[70]-dsAlk(c)	CAGGCCCCAGCCACGCTGAGAGCCAGCAGAAATCTACGcgacaaggcagcga
CAS5	PAS3-dsAlk	/5Hexynyl/ctacgttcattcgctgccttgtcg
Blockers1	57[137]-59[173]	GGTGGATTGACGGATTCTCCGTGGttGCGAACG
Blockers1	1[148]-18[140]	TAAAGTTttttttTCTCAGGCGGATAttttttttttttttttttttttttttttGCGG
Blockers1	12[169]-14[140]	CGTTAGTttttttAAACATAGCCCCttttttttttttttttttttttttttttCAGC
FRET1	50[121]-49[115]	TAATTGAATCTGAGTAGAAGAACTCAAA
FRET1	25[53]-27[45]	ACCAATATATTTTAGTTTTCC
FRET1	45[32]-43[20]	GAACAGGGCGATGGCttttttttttttttttttttttttttttTTAGT
FRET1	33[24]-19[38]	GGATTCGCCTGATTGCTTGAATTATTTCC
FRET1	27[117]-25[132]	AATCATAGGGTTATATTTTAGTATCATATG
FRET1	30[144]-21[143]	GAGGCCCGCttttttttttttttttttttttttttttAAACAAAGTCAGA
FRET1	42[140]-69[148]	TAACCCTCGTTTTGCCAAAAAGTAGTCAGTTTAGAATTATTTCAACGCAAGG
FRET1	49[41]-50[52]	GGTACGCCAACGCTCGCAGATTCACCAttGGGT
FRET2	50[121]-(3')Cy3	TAATTGAATCTGAGTAGAAGAACTCAAA/3Cy3/
FRET2	25[53]-27[45]	ACCAATATATTTTAGTTTTCC
FRET2	45[32]-43[20]	GAACAGGGCGATGGCttttttttttttttttttttttttttttTTAGT
FRET2	33[24]-19[38]	GGATTCGCCTGATTGCTTGAATTATTTCC
FRET2	27[117]-(5')Cy5	/5Cy5/AATCATAGGGTTATATTTTAGTATCATATG
FRET2	30[144]-21[143]	GAGGCCCGCttttttttttttttttttttttttttttAAACAAAGTCAGA
FRET2	42[140]-69[148]	TAACCCTCGTTTTGCCAAAAAGTAGTCAGTTTAGAATTATTTCAACGCAAGG
FRET2	49[41]-50[52]	GGTACGCCAACGCTCGCAGATTCACCAttGGGT
Opening Key	OpK1	atgctcctaATCCTACACTGATGTTTA; (stem-loop colour code)<br>atgctcctaATCCTACACTGATGTTTA (double-helix colour code)
Opening Key	OpK2	gttgtggagTGTCAGTTTGTCAAATAC
Opening Key	OpK3	aatatctaaCAACGGAATCCCAAAAGC
Opening Key	OpK4	gacttagaaTAGCTTATCAGACTGAT
Opening Key	OpK5	tgttaagatCTGTGCGTGTGACAGCG

Opening Key	OpK6	aattcggccAACATTCAACGCTGTCGG
Opening Key	OpK7	aacttcgtaTAATCGTGATTTAGCATT
Closing Key	ClK1	ACATCAGTGTAGGATtaggagcat; (stem-loop colour code)<br>ACATCAGTGTAGGATtaggagcat (double-helix colour code)
Closing Key	ClK2	TGACAAACTGACActccacaac
Closing Key	ClK3	TTGGGATTCCGTTGttagatatt
Closing Key	ClK4	GTCTGATAAGCTAttctaagtc
Closing Key	ClK5	GTCACACGCACAGatcttaaca
Closing Key	ClK6	CAGCGTTGAATGTTggccgaatt
Closing Key	ClK7	GCTAAATCACGATTAtacgaagtt
Unspecific Key	UnK	GCATGCCGCTGCGCTTGGCCGCGAAAAC
InvaderA	Eco ssDNA	CTAAATACTACATACGAGgaaggatatcaggc
InvaderB	Eco DBell	CCGCGCCCTCGTTTTTACGAGGGGCAGCAGTTTTCTGCTGCCGCGGCATACGAGg aaggatatcaggc
InvaderC	Eco Biotin	/Biotin/CTAAATACTACATACGAGgaaggatatcaggc

Supplementary References.

1 Stahl, E., Martin, T. G., Praetorius, F. & Dietz, H. Facile and scalable preparation of pure and dense DNA origami solutions. *Angew Chem Int Ed Engl* **53**, 12735-12740, doi:10.1002/anie.201405991 (2014).

2 Yang, Y. R., Liu, Y. & Yan, H. DNA Nanostructures as Programmable Biomolecular Scaffolds. *Bioconjug Chem* **26**, 1381-1395, doi:10.1021/acs.bioconjchem.5b00194 (2015).
